# Unprecedented deoxygenation at C-7 of the ansamitocin core during mutasynthetic biotransformations

**DOI:** 10.3762/bjoc.8.96

**Published:** 2012-06-11

**Authors:** Tobias Knobloch, Gerald Dräger, Wera Collisi, Florenz Sasse, Andreas Kirschning

**Affiliations:** 1 Institute of Organic Chemistry and Center of Biomolecular Drug Research (BMWZ), Leibniz University Hannover, Schneiderberg 1b, 30167 Hannover, Germany; 2 Department of Chemical Biology, Helmholtz Center for Infectious Research (HZI), Inhoffenstraße 7, D-38124 Braunschweig, Germany

**Keywords:** ansamitocins, antibiotics, antitumor agents, mutasynthesis, natural products

## Abstract

We describe the unprecedented formation of six ansamitocin derivatives that are deoxygenated at C-7 of the ansamitocin core, obtained during fermentation experiments by employing a variety of *Actinosynnema pretiosum* mutants and mutasynthetic approaches. We suggest that the formation of these derivatives is based on elimination at C-7/C-8 followed by reduction(s) of the intermediate enone. In bioactivity tests, only ansamitocin derivatives bearing an ester side chain at C-3 showed strong antiproliferative activity.

## Introduction

Natural products still play an important role as lead structures for the treatment of infectious diseases and cancer. However, natural products have lost some of their attraction for the development of pharmaceuticals because of their structural complexity and the difficulties associated with accessing analogues for structure–activity relationship studies. Total synthesis approaches are still a tour de force and are hardly employed, while commonly semisynthesis as well as biotechnological approaches are widely pursued in industrial research [1–3]. Investigations into the biosynthesis of natural products have not only allowed us to understand the synthetic principles that nature pursues, but have also provided tools, mainly based on genetic engineering, which can be exploited in natural product synthesis [4].

Producer strains genetically blocked in the biosynthesis of important and complex natural products can serve as such new tools. The synthetic concept based on these blocked mutants is termed “mutational biosynthesis”, or in short mutasynthesis, and it relies on the cellular uptake of modified biosynthetic intermediates, sometimes termed mutasynthons, and their incorporation into complex secondary metabolites [5–7].

When making use of mutants that are blocked in early stages of a given biosynthesis pathway, the concept of mutasynthesis may be compared to a (partial) natural product total synthesis. When further modification of an advanced biosynthetic intermediate with an established core structure towards bioactive natural products and analogues is conducted, mutasynthesis may be regarded as the “endgame” of a total synthesis [4].

The ansamitocins (maytansinoids) **3**–**5** are ideally suited for mutasynthetic modifications and the creation of new analogues because they are highly potent antitumor active compounds that inhibit the growth of different leukemia cell lines as well as human solid tumors at very low concentrations (10^−3^ to 10^−7^ µg/mL) [8]. In contrast to colchicine, maytansinoids such as ansamitocins bind to β-tubulin monomers at a site overlapping the vinca alkaloid binding site [9].

**Scheme 1:**
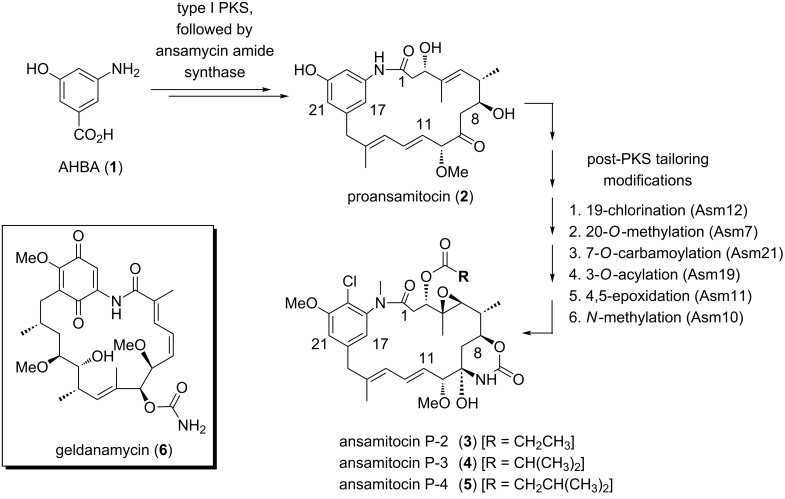
Summary of ansamitocin biosynthesis and structure of the related ansamycin antibiotic geldanamycin (**6**).

Recently, we disclosed several mutasynthetic studies aimed at the production of derivatives of ansamitocins **3**–**5** [10–12] as well as of geldanamycin (**6**), utilizing mutant strains of *Actinosynnema pretiosum*, the ansamitocin producer [13–17], and *Streptomyces hygroscopicus*, the geldanamycin producer [18–19]. These engineered strains are unable to biosynthesize 3-amino-5-hydroxybenzoic acid (**1**) [20], the common starter unit for both polyketide synthases (PKS) (Scheme 1). These assembly-line-type multienzymes are responsible for setting up the complete carbon backbone of both ansamycin antibiotics [21–24].

More precisely, the biosynthesis of ansamitocins relies on a type I modular polyketide synthase (PKS), with 3-amino-5-hydroxybenzoic acid (**1**, AHBA) [20] as the starter unit followed by chain extension by one “glycolate”, three propionate and three acetate units. The last PKS module holds *seco*-proansamitocin, which is released and cyclized, presumably by an ansamycin amide synthase (Asm9) [21–24], to yield the 19-membered macrocyclic lactam proansamitocin (**2**). Proansamitocin (**2**) is transformed into bioactive compounds **3**–**5** by a set of post-PKS tailoring steps, following a predetermined, only partly flexible logic (Scheme 1) [16].

**Figure 1:**
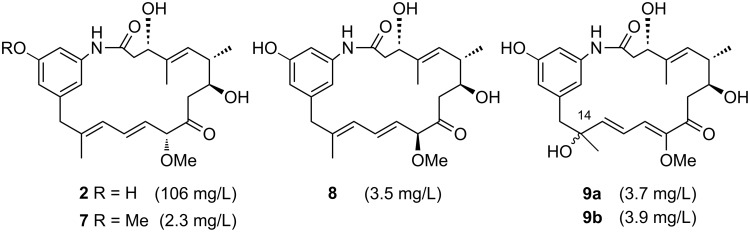
Fermentation products, proansamitocin (**2**) and derivatives **7**–**9**, of the Asm12 and Asm21-blocked (chlorination, carbamoylation) mutant strain *A. pretiosum* Δasm12/21 (yields given as isolated product per volume of fermentation broth) [17].

Complementing our studies with mutant strain *A. pretiosum* HGF073, blocked in the biosynthesis of the PKS starter unit AHBA **1** [13–16], we recently reported the use of a mutant of *A. pretiosum* blocked in Asm12 (chlorination) and Asm21 (carbamoylation) and therefore producing proansamitocin (**2**) in good yield (up to 106 mg/L of fermentation broth) [17]. Additionally, we isolated small amounts of *O*-methyl proansamitocin **7** (2.3 mg/L), 10-*epi*-proansamitocin **8** (3.5 mg/L) and two diastereomeric byproducts **9a** and **9b** (7.6 mg/L; 1:1 ratio) from the fermentation broth of *A. pretiosum* Δasm12/21 (Figure 1). We also showed that none of these proansamitocin derivatives exhibit antiproliferative activity.

Herein, we describe the unprecedented formation of ansamitocin derivatives that are deoxygenated at C-7 of the ansamitocin core, obtained by us during fermentation experiments using a variety of *A. pretiosum* mutants and mutasynthetic approaches.

## Results and Discussion

**Scheme 2:**
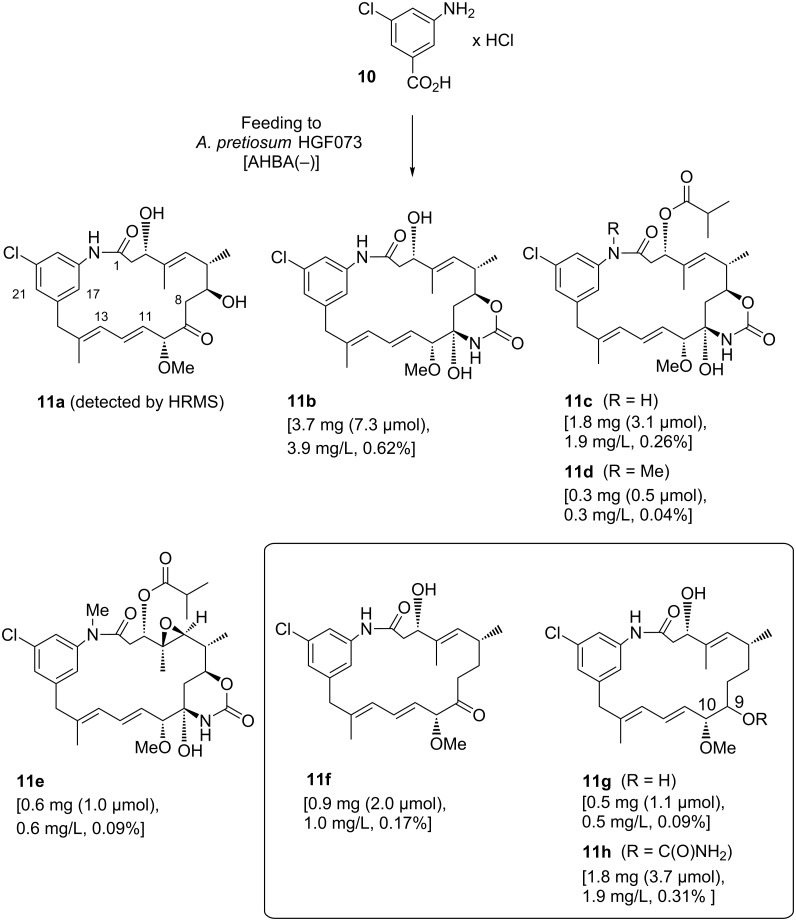
Mutasynthetic preparation of ansamitocin derivatives **11a**–**h** by using 3-amino-5-chlorobenzoic acid (**10**) as a mutasynthon (yields in % are calculated with reference to the amount of mutasynthon **10** fed).

In the course of our well-established mutasynthesis experiments with *A. pretiosum* HGF073, a mutant that is unable to produce the essential starter unit AHBA (**1**) by itself, we achieved the generation of several novel ansamitocin derivatives, among other things, based on the simple mutasynthon 3-amino-5-chlorobenzoic acid (**10**, Scheme 2).

With the exception of proansamitocin analogue **11a**, all new metabolites were isolated on a preparative scale and fully characterized. These compounds correspond to the known stepwise sequence of postketide synthase tailoring transformations, and those products and similar compounds have been reported by us before [16]. As evident from the variety of compounds isolated, proansamitocin analogues resulting from supplementation of AHBA analogue **10** to *A. pretiosum* HGF073 were not efficiently processed by the enzymes involved in post-PKS tailoring. In addition to the ordinary compounds **11a**–**e**, the experiment yielded three compounds of an unprecedented type (**11f**–**h**) whose appearance could not be attributed to the known tailoring transformations. Herein, we now describe for the first time the isolation and characterization of compounds **11f**–**h** sharing, in contrast to all other (pro)ansamitocin derivatives known so far, the common feature of deoxygenation at C-7. In addition, proansamitocin derivatives **11g**–**h** are notable for their C-9 alcohol, while **11h** shows additional carbamoylation of the unusual alcohol moiety. The extraordinary proansamitocin derivatives **11f**–**h** were fully characterized, except for the configuration at C-9 in **11g** and **11h** (single diastereomer). Overlap with other signals in the ^1^H NMR spectra hampered complete assignment of all coupling constants at C-9 except for *J*_9,10_ = 7.2 Hz, which, however, is not diagnostic.

**Scheme 3:**
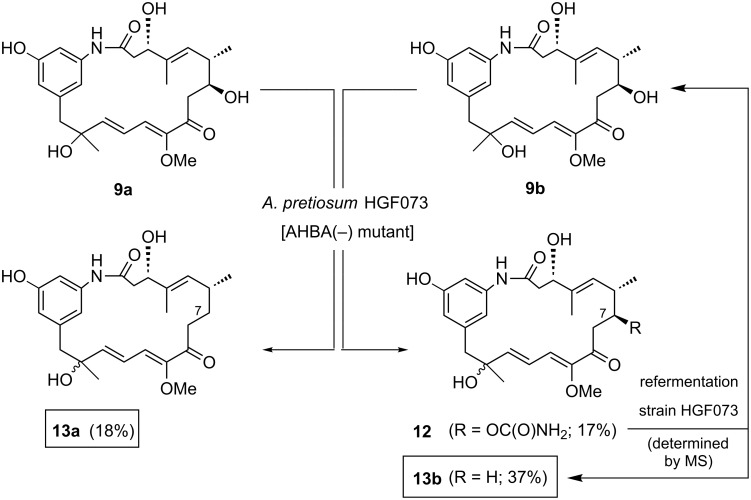
Mutasynthetic biotransformation of proansamitocin derivatives **9a** and **9b** with AHBA(−) mutant *A. pretiosum* HGF073 (starting materials could be reisolated: **9a**: 77% reisolated, **9b**: 23% reisolated).

In continuation of our experiments with advanced biosynthetic intermediates, such as proansamitocin (**2**) [25] and *seco*-acid derivatives [26–27] serving as mutasynthons in experiments with early-stage-blocked mutants, we also tested the unusual metabolites **9a** and **9b** [17] as precursors for further processing by the AHBA(−)-mutant of the ansamitocin producer (Scheme 3).

Originally obtained by fermentation of a mutant blocked to the greatest extent in the post-PKS transformation sequence (*A. pretiosum* Δasm12/21) [17], it was questionable whether these compounds, differing substantially from proansamitocin (**2**) both by a rearranged diene system and an alcohol moiety at C-14, would be accepted by the tailoring enzymes. Surprisingly, when the rearranged oxidation products **9a** and **9b** were each supplemented to cultures of *A. pretiosum* HGF073, new products were formed. While the carbamoylated derivative **12** resulting from biotransformation of alcohol **9b** can be attributed to the activity of the carbamoyltransferase Asm21, thereby providing an indication pertaining to its substrate flexibility, products **13a** and **13b** obtained from both experiments are more unusual and differ from the starting material by being deoxygenated at C-7.

However, the structure of compound **12** strongly contrasts with all of the other 7-*O*-carbamoylated (pro)ansamitocin derivatives that we have obtained with this kind of feeding experiment so far [17]. In the case of compound **12**, the carbinolamide moiety is not present in a cyclic halfaminal form (δ_C-9_ ~197 ppm instead of ~82 ppm), despite the fact that the keto group at C-9 is still present. This result may be attributed to the keto group at C-9 being in conjugation with the diene system, resulting in a reduced activity of the carbonyl group and an alteration of the macrolactam ring conformation.

We based the determination of deoxygenation at C-7 in **13a** and **13b** on MS data and the appearance of a secondary carbon atom in exchange of the tertiary carbinol at C-7 on phase-sensitive ^1^H–^13^C-correlation NMR spectra (HSQC). It needs to be noted that NOE-analysis combined with molecular modeling did not allow elucidation of the absolute configuration at C-14 for the new derivatives **13a** and **13b**, as it did not allow for the starting compounds **9a** and **9b** [17].

In order to shed light on the sequence of events leading to deoxygenation, the carbamoylated derivative **12** was again fed to a culture of *A. pretiosum* HGF073, and the formation of new products was analyzed by UPLC-HRMS of the partially purified crude extract (Scheme 3). Indeed, the expected deoxygenated product **13b** could be detected, but was also accompanied by the formation of alcohol **9b**. Compound **9b** may either have resulted from Michael addition of water to the suspected intermediate enone **17** (see later in Scheme 5) or from hydrolytic cleavage of the carbinolamide **12**.

**Scheme 4:**
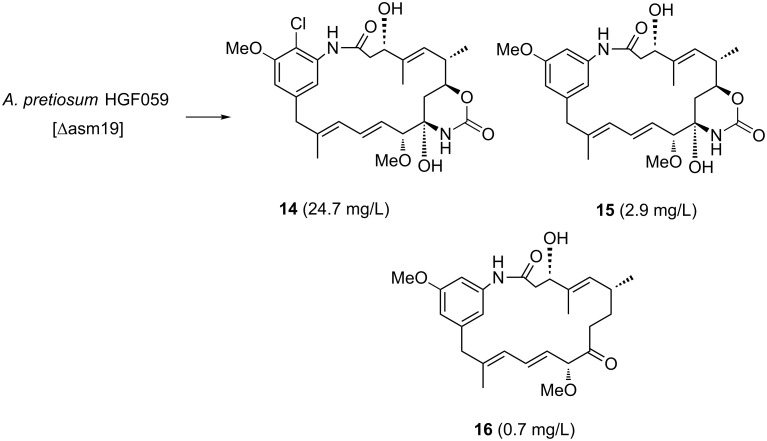
Fermentation products **14**–**16** of acyl transferase Asm19-blocked mutant *A. pretiosum* HGF059 (Δasm19) (yields given as isolated product per volume of fermentation broth).

A blocked mutant of *A. pretiosum* that is unable to carry out acylation of the C-3 alcohol of the carbamoylated proansamitocin derivative precursor due to genetic inactivation of the acyl transferase Asm19, was reported by Floss et al. [21,28]. It was known that mutant strain *A. pretiosum* HGF059 produces the expected ansamitocin derivative **14** in good yield (Scheme 4) [28]. Besides the ester side chain, compound **14** also lacks the epoxy functionality and the *N*-methyl group. These two tailoring steps finalize the biosynthesis of ansamitocin P-3 (AP-3, **4**) and occur only after acylation has taken place (Scheme 1).

While examining the fermentation extract for byproducts we were able to identify two new metabolites, **15** and **16**. Formation of compound **15** can be traced back to inefficient chlorination, a phenomenon that we have encountered before in other feeding experiments with proansamitocin [25]. More unusual is metabolite **16,** which is yet another example of a case in which deoxygenation at C-7 has taken place.

**Scheme 5:**
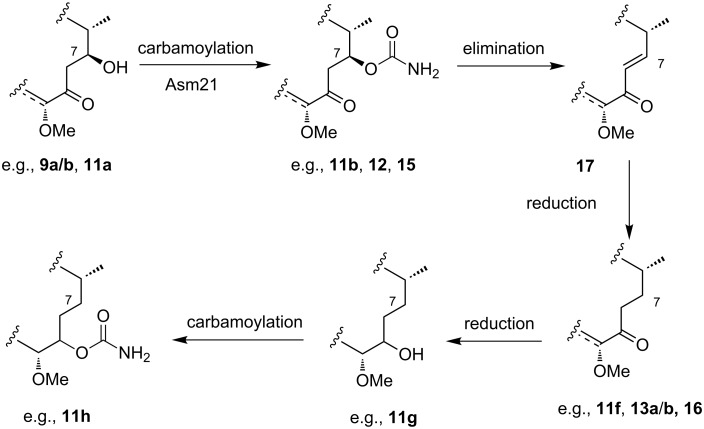
Possible mechanism of deoxygenation at C-7 of proansamitocin derivatives.

In summary, deoxygenation may occur when the AHBA-blocked mutant *A. pretiosum* HGF073 or the acyl transferase Asm19-blocked mutant *A. pretiosum* HGF059 are employed, whereas the reduction process does not occur with the blocked mutant *A. pretiosum* ∆asm12/21. The major difference between these three mutants is the presence (HGF073, HGF059) or absence (Δasm12/21) of the carbamoyl transferase Asm21. Carbamoylation of proansamitocin derivatives at C-7 (e.g. **11a**, **9a**/**b** and **7**) introduces a fairly good leaving group β-positioned to the keto group, thereby facilitating elimination to enones **17** (Scheme 5).

In the case of compound **12,** which predominantly exists in a form lacking the typical cyclized carbinolamide moiety, the C-9 keto group can preserve its electronic properties, thus facilitating the elimination step. Finally, the activity of a tailoring reductase, which in all likelihood is not part of the PKS, catalyzes the reduction of the α,β-unsaturated bond, yielding the deoxygenated derivatives (**11f**, **13a/b**, **16**). Diastereoselective reduction of the C-9 keto group and carbamoylation of intermediate **11f** would then result in compounds **11g** and **11h**, respectively.

A factor contributing to the formation of deoxygenated proansamitocin derivatives is likely the usage of the carbamoylated precursors by the Asm19 acyl transferase. Acylation is a crucial bottleneck step in the otherwise partly flexible sequence of post-PKS transformations. When this step cannot occur due to the absence of an active acyl transferase (as in *A. pretiosum* HGF059), or if the transformation is inefficient (e.g., compound **11b**) or even nonexistent due to an unusual substrate (e.g., compound **12**), carbamoylated intermediates accumulate. Indeed, the carbamoylated product **11b** was the major product of the mutasynthesis experiment with mutasynthon **10**. The carbamoylated compounds may then be channeled into the pathway leading to deoxygenated products. Depending on the substrate, this process may be quite efficient. For instance, no carbamoylated product could be isolated after the biotransformation of compound **9a**, indicating an efficient transformation of the suspected carbamoylated intermediate to the final product **13a**.

All (pro)ansamitocin derivatives fully characterized by NMR were also subjected to in vitro biological testing with different human cell lines derived from tumors or the umbilical vein. The results from these tests are given as values for the half-maximal inhibitory concentration of the respective ansamitocin derivatives in comparison to the “gold standard” ansamitocin P-3 (**4**, Table 1). As expected [14], all (pro)ansamitocin derivatives lacking the ester side chain at C-3 (**11b**, **11g**–**h**, **12**–**16**) do not show any antiproliferative activity (IC_50_ > 800 nM) for at least two of the cell lines listed in Table 1.

**Table 1:** Antiproliferative activity IC_50_ [nmol/L] of **11c**–**e** in comparison to AP-3 (**4**).^a^

compound	cell line					

	U-937	A-431	SK- OV-3	PC-3	MCF-7	HUVEC

AP-3 (**4**)	0.01	0.08	0.05	0.06	n.d.	0.02

**11c**	0.5	1.58	0.66	0.30	0.90	0.32
**11d**	0.05	0.10	0.05	0.16	0.11	0.08
**11e**	0.18	0.35	0.21	0.53	0.41	0.21

^a^Values shown are means of two determinations in parallel; human cell lines: U-937 (histiocytic lymphoma), A-431 (epidermoid carcinoma), SK-OV-3 (ovary adenocarcinoma), PC-3 (prostate adenocarcinoma), MCF-7 (breast adenocarcinoma), HUVEC (umbilical vein endothelial cells); n.d. = not determined.

Compounds **11c**–**e**, bearing the ester side chain at C-3, predominantly showed activities in the pM range. As seen also with AP-3 (**4**)**,** there is no significant difference between cancerous and healthy cells. The most active compound was the *N*-methyl derivative **11d**, which reached the activity of the standard AP-3 (**4**) for selected cell lines.

## Conclusion

In conclusion, we disclose the isolation and chemical and antiproliferative activity characterization of several novel ansamitocin derivatives that are deoxygenated at C-7. We used three different *A. pretiosum* mutants and a variety of mutasynthetic approaches. These preliminary studies on the deoxygenation at C-7 suggest that it occurs by elimination at C-7/C-8 after carbamoylation has taken place, followed by reduction mediated by an unknown reductase, which is not part of the main assembly line PKS. As expected, all ansamitocin compounds bearing the ester side chain at C-3 predominantly showed activities in the pM range.

## Experimental

Analytical details are given in the Supporting Information File 1.

### Cultivation

In general, cultivation of microbial strains on agar plates was conducted in a Heraeus incubator at 30 °C, while cultivation in a shake flask was performed in a multilevel New Brunswick Scientific Innova 4900 gyratory multi-shaker at 200 rpm at 29 °C.

Unless otherwise noted, the cultivation media were prepared using distilled water and sterilized by autoclaving: YMG medium – 10 g/L malt extract (Sigma), 4 g/L yeast extract (Bacto), 4 g/L D(+)- glucose∙H_2_O; YMG agar – YMG medium plus 22 g/L agar (Bacto); K-medium [29], basal composition (final start concentration in the main culture corresponds to 5/6 of the values given for this medium due to dilution) – 60 g/L dextrin from maize starch (Fluka), 30 g/L D(+)-maltose∙H_2_O (Fluka), 5.25 g/L cottonseed flour (Proflo), 5 g/L CaCO_3_, 4.5 g/L yeast extract (Bacto), 300 mg/L K_2_HPO_4_ (Fluka, TraceSelect), 2 mg/L FeSO_4_∙7H_2_O. K-medium, additive – autoclaved and added separately: 3 g/L L-valine (final start concentration in the main culture, from a 3% (w/v) stock solution). *A. pretiosum* HGF073 is a replicate of strain HGF056 reported in [22].

### Fermentation of *A. pretiosum* strains

*A. pretiosum* strains (HGF073, HGF059, Δasm12/21) were stored as spore suspensions in 40% (v/v) glycerol/water at −80 °C, and used for the inoculation of YMG agar plates. Following incubation of the plates for 4 d at 30 °C, 5–8 well-sporulated colonies were transferred to a 1.5 mL tube charged with 1 mL of sterile distilled water and filled to approx. 50% height with sterile glass beads (Ø = 2 mm, washed with dilute hydrochloric acid). After vortex-mixing, the resulting suspension was used for the inoculation of precultures in bottom-baffled 250 mL Erlenmeyer flasks charged with YMG medium (50 mL per flask, with additional steel spring). Precultures were shaken for 2 d at 29 °C before inoculation of main production cultures (1/15 dilution). Cultivations were performed in K-medium with additives – 42 mL K-medium with L-valine, 3 mL preculture and one drop of SAG 471 anti-foam (GE Bayer Silicones) [30] – by using nonbaffled 250 mL Erlenmeyer flasks (final volume: 35–60 mL per flask, with additional steel spring). Shaking was continued at 29 °C for a total cultivation time of 7–10 days.

For detection of novel products from test cultures, samples of the culture broth (200 μL) were mixed with ethanol (200 μL), centrifuged (20800*g*, 3 min, 4 °C) and the clear supernatant subjected to UPLC-ESIMS analysis. Failing detection of novel products, the culture broth was extracted three times with ethyl acetate, dried over MgSO_4_, concentrated in vacuo, and filtered over silica gel with ethyl acetate, and the solvent was removed in vacuo. The residue was dissolved in methanol (1 mL) and subjected to UPLC-ESIMS analysis.

For isolation of novel products from (large-scale) fermentations, the combined fermentation broth was extracted with ethyl acetate as described above, and the crude extract subjected to a sequence of chromatographic purifications (Supporting Information File 1).

### Mutasynthesis with *A. pretiosum* HGF073

In mutasynthesis experiments with *A. pretiosum* HGF073, production cultures were shaken for 2 d after inoculation (see above) before mutasynthons were added (**9a**, **9b**, **10** or **12**). For novel mutasynthons, productivity of the strain was first monitored by parallel feeding of mutasynthons for which acceptance was known (e.g., the natural starter building block: 3-Amino-5-hydroxybenzoic acid, hydrochloride salt (**1**)). Mutasynthons were dissolved in DMSO/water [preferably 1:1; volume of feeding solution not exceeding 10% (v/v) with respect to the recipient culture] and sterilized by filtration.

Mutasynthon **10** (1.25 mmol/L of fermentation broth) was added to production cultures with a combined volume of 945 mL continuously (drop-wise) over the time-course of 3.5 d, by using autoclavable, syringe pump-driven feeding capillaries – Braintree Scientific BS-9000-8 syringe pump with Upchurch Scientific high-purity Teflon^®^ PFA tubing (1/16” OD, 0.1” ID) and Tefzel^®^ connectors.

Biotransformation of the proansamitocin derivatives **9a**, **9b** and **12** was carried out by supplementing a production culture of *A. pretiosum* HGF073 (45 mL final volume; K-medium; see above) with the respective derivative [**9a/b** (each*:* 4.5 mg, 9.8 µmol, dissolved in 2 mL DMSO:H_2_O = 1:1); **12** (0.1 mg, 0.2 µmol, dissolved in 1 mL DMSO)] in a single portion after 2.5 (for **12**) to 3.5 d (for **9a/b**) of shaking.

Following biotransformation (refermentation) of proansamitocin derivative **12**, only UPLC-ESIMS analysis of the silica-gel-filtered ethyl acetate extract taken up in methanol (see above) was carried out. The retention times and mass spectra of the detected product derivatives **9b** and **13b** were identical to those of the previously isolated materials (Supporting Information File 1).

## Supporting Information

The supporting information provides purification protocols of fermentations, a short description of the cell proliferation assay, analytical descriptions of new metabolites and copies of ^1^H and ^13^C NMR spectra.

File 1 Analytical details and compound spectra.
