# Supplementary material for: Unprecedented deoxygenation at C-7 of the ansamitocin core during mutasynthetic biotransformations
Source: Beilstein J Org Chem. 2012 Jun 11;8:861–9. doi: 10.3762/bjoc.8.96 (PMC3388874; doi:10.3762/bjoc.8.96)
Supplement: File 1 — Analytical details and compound spectra. [file Beilstein_J_Org_Chem-08-861-s001.pdf]

Supporting Information

for

Unprecedented deoxygenation at C-7 of the ansamitocin core during mutasynthetic biotransformations

Tobias Knobloch[1], Gerald Dräger[1], Wera Collisi[2], Florenz Sasse[2] and Andreas Kirschning[*1]

Address: [1]Institute of Organic Chemistry and Center of Biomolecular Drug Research (BMWZ), Leibniz University Hannover, Schneiderberg 1b, 30167 Hannover, Germany and [2]Department of Chemical Biology, Helmholtz Center for Infectious Research (HZI), Inhoffenstraße 7, D-38124 Braunschweig, Germany

Email: Andreas Kirschning[*] - andreas.kirschning@oci.uni-hannover.de

* Corresponding author

Analytical details and compound spectra

1. General Information S2
2. Purification protocols and analytical data S3
   2.1 20-Chloro-proansamitocin derivatives **11f–h** S4
   2.2 $\Delta^{10,12}$-Proansamitocin derivatives **12**, **13a**, **13b** S7
   2.3 20-*O*-Methyl-proansamitocin derivatives **14–16** S10
3. Cell proliferation assay S12
4. NMR spectra S13
5. References S22

1. General Information

^{1}H NMR spectra were recorded at 400 MHz with a Bruker Avance-400 or at 500 MHz with a Bruker DRX-500 spectrometer at 283 or 323 K. ^{13}C NMR spectra were recorded at 100 MHz with a Bruker Avance-400 and at 125 MHz with a Bruker DRX-500 instrument. Multiplicities are described using the following abbreviations: s = singlet, d = doublet, t = triplet, q = quartet, m = multiplet, b = broad. Chemical shift values of ^{1}H and ^{13}C NMR spectra are commonly reported in ppm relative to the residual solvent signal as the internal standard [S1]. The multiplicities refer to the resonances in the off-resonance decoupled spectra and were elucidated by using phase-sensitive HSQC experiments. Multiplicities are reported by using the following abbreviations: s = singlet (due to quaternary carbon), d = doublet (methine), q = quartet (methyl), t = triplet (methylene). The interpretation of the NMR spectra of ansamitocin derivatives required the performance of ^{1}H–^{1}H correlation (COSY) and ^{1}H–^{13}C correlation (phase-sensitive HSQC, HMBC) experiments. Mass spectra were alternatively obtained with a type VG Autospec (EI) spectrometer at 75 eV (Micromass), a type LCT (ESI) (Micromass) equipped with a lockspray dual ion source in combination with a WATERS Alliance 2695 LC system, or with a type Q-TOF premier (Micromass) spectrometer (ESI mode) in combination with a Waters Acquity UPLC system equipped with a Waters Acquity UPLC BEH C18 1.7 µm (SN 01473711315545) column – solvent A: water + 0.1% (v/v) formic acid, solvent B: MeCN or MeOH (given in experimental part) + 0.1% (v/v) formic acid; flow rate = 0.4 mL/min; gradient (*t* [min]/solvent B [%]): (0/5) (2.5/95) (6.5/95) (6.6/5) (8/5); retention times (t_R) given in the experimental part. Ion mass signals (*m*/*z*) are reported as values in atomic mass units. Analytical thin-layer chromatography was performed by using precoated silica gel 60 F_{254} plates (Merck, Darmstadt) and the spots were visualized with UV light at 254 nm or alternatively by staining with ninhydrin,

permanganate or 4-methoxybenzaldehyde solutions [S2]. Flash column chromatography was performed on Machery–Nagel silica gel (particle size = 40–63 μm). Size exclusion chromatography was performed with Sephadex® LH-20 stationary phase (500 × Ø20 mm) and methanol as eluent. Isolation of ansamitocin derivatives was achieved by preparative high-performance liquid chromatography using a Merck Hitachi LaChrom system, pump L-7150, interface D-7000, diode array detector L-7450 (λ = 220–400 nm, preferred monitoring at λ = 248 nm) with columns (abbreviations referred to in the experimental part are given in parentheses): (C18-$P_{[A]}$) TrentecReprosil-Pur 120 C18 AQ 5 μm, 250 mm × 25 mm, with guard column, 30 mm × 20 mm; (C18-SP) Trentec Reprosil-Pur 120 C18 AQ 5 μm, 250 mm × 8 mm, with guard column, 40 mm × 8 mm; (CN-SP) Trentec Reprosil 100 CN 5 μm, 250 mm × 8 mm, with guard column, 40 mm × 8 mm. Alternatively, preparative high-performance liquid chromatography was performed by using a Varian system, pump Prepstar Model 218, variable wavelength detector Prostar (λ = 248 nm) with parallel mass spectrometric detection (Micromass type ZMD ESI-Quad spectrometer) using the stationary phase C18-$P_{[A]}$, indicated by abbreviation C18-$P_{[B]}$. Operating conditions and retention times (t_R) are reported in the experimental part.

Melting points were measured by using either a SRS OptiMelt apparatus or an Electrothermal IA 9200 instrument and are reported uncorrected. Commercially available reagents, chromatography type or dry solvents were used as received or purified by standard techniques according to the literature [S2].

2. Purification protocols and analytical data

Analytical data for proansamitocin (**2**) and derivatives **7**–**9a**/**b** was reported before [S3]. Likewise, 20-chloro ansamitocin derivatives **11a**–**11e** were described in [S4].

2.1 20-Chloro-proansamitocin derivatives 11f–h

Isolation of mutaproducts from large-scale fermentation was achieved by combining several fermentation broths, which were extracted with ethyl acetate, and the crude extract was subjected to a sequence of chromatographic purifications (Table S1).

Table S1: Chromatographic purification of proansamitocin derivatives **11f**–**h**.

Sample	column	conditions	fractions
crude extract	SiO_2	petroleum ether:ethyl acetate 4:1 → ethyl acetate	F-1 (R_f (EE) 0.6–0.05)
F-1	C18-P[A]	H_2O:MeOH [A:B], flow rate = 5.0 mL/min gradient (*t* [min]/B [%]): (0/20) (5/20) (60/60) (120/100)	F-2 (t_R = 104.0–115.0 min)
F-2	CN-SP	H_2O:MeCN [A:B], flow rate = 2.5 mL/min gradient (*t* [min]/B [%]): (0/5) (5/5) (45/30) (55/35) (85/45)	F-3 (t_R = 61.0–63.5 min) F-4 (t_R = 63.5–66.0 min and 72.0–74.5 min) F-5 (t_R = 66.5–69.0 min)
F-3	C18-SP	H_2O:MeCN [A:B], flow rate = 2.5 mL/min gradient (*t* [min]/B [%]): (0/5) (5/5) (45/30) (55/35) (85/45) (90/50) (90.1/100) (100/100)	**11f** (t_R = 86.0 min)
F-4	C18-SP	H_2O:MeCN [A:B], flow rate = 2.5 mL/min gradient (*t* [min]/B [%]): (0/5) (5/5) (45/30) (55/35) (85/45) (90/50) (90.1/100) (100/100)	**11g** (t_R = 89.5 min)
F-5	C18-SP	H_2O:MeCN [A:B], flow rate = 2.5 mL/min gradient (*t* [min]/B [%]): (0/5) (5/5) (45/30) (55/35) (85/45) (90/50) (90.1/100) (100/100)	**11h** (t_R = 93.5 min)

The derivatives **11f** (0.9 mg, 2.0 µmol, 1.0 mg/L), **11g** (0.5 mg, 1.1 µmol, 0.5 mg/L) and **11h** (1.8 mg, 3.7 µmol, 1.9 mg/L) were obtained as colorless solids. All fractions (**11b**–**h**) gave a total yield of 18.7 µmol, which corresponds to 1.6% with respect to aminobenzoic acid derivative **10** employed in the mutasynthetic experiment.

7-Deoxy-20-chloro-proansamitocin (11f)

^{1}H NMR (500 MHz, methanol-d_4, CHD_2OD = 3.31 ppm) δ 7.38 (dd, *J* = 1.7, 1.7 Hz, 1H, 17-H), 7.09 (dd, *J* = 1.7, 1.7 Hz, 1H, 19-H), 7.00 (dd, *J* = 1.7, 1.7 Hz, 1H, 21-H), 6.81 (dd, *J* = 15.1, 11.2 Hz, 1H, 12-H), 6.07 (d, *J* = 11.2 Hz, 1H, 13-H), 5.37 (dd, *J* = 15.1, 7.8 Hz, 1H, 11-H), 5.09 (dq, *J* = 8.4, 1.2 Hz, 1H, 5-H), 4.47 (d, *J* = 7.8 Hz, 1H, 10-H), 4.32 (dd, *J* = 10.3, 4.7 Hz, 1H, 3-H), 3.36 (d, *J* = 13.4 Hz, 1H, 15-H_a), 3.32 (s, 3H, 10-OMe), 3.21 (d, *J* = 13.4 Hz, 1H, 15-H_b), 2.62 (dd, *J* = 12.5, 4.7 Hz, 1H, 2-H_a), 2.62–2.55 (m, 1H, 8-H_a), 2.56 (dd, *J* = 12.5, 10.3 Hz, 1H, 2-H_b), 2.31 (ddd, *J* = 16.0, 6.0, 5.8 Hz, 1H, 8-H_b), 2.00–1.95 (m, 1H, 6-H), 1.86–1.77 (m, 1H, 7-H_a), 1.71 (s, 3H, 14-Me), 1.63 (d, *J* = 1.2 Hz, 3H, 4-Me), 1.23–1.15 (m, 1H, 7-H_b), 0.48 (d, *J* = 6.5 Hz, 3H, 6-Me) ppm; ^{13}C NMR (125 MHz, methanol-d_4, methanol-d_4 = 49.0 ppm) δ 209.7 (s, C-9), 171.0 (s, C-1), 143.6 (s, C-16), 141.4 (s, C-14), 135.2 (s, C-4), 134.5 (s, C-20), 134.0 (d, C-5), 133.3 (d, C-12), 126.5 (d, C-13), 126.1 (d, C-11), 125.5 (d, C-21), 119.8 (d, C-19), 119.6 (d, C-17), 88.8 (d, C-10), 75.7 (d, C-3), 56.7 (q, 10-OMe), 46.3 (t, C-15), 44.2 (t, C-2), 36.2 (t, C-8), 32.1 (t, C-7), 31.5 (d, C-6), 19.4 (q, 6-Me), 16.3 (q, 14-Me), 11.4 (q, 4-Me) ppm, the quaternary atom C-18 could not be detected; UPLC-MS [MeCN] t_R 2.04 min; HRMS-ESI (*m*/*z*): $[M + H]^+$ calcd for $C_{25}H_{33}ClNO_4$ 446.2098; found: 446.2102.

7-Deoxy-9-hydro-20-chloro-proansamitocin (11g)

^{1}H NMR (500 MHz, methanol-d_4, CHD_2OD = 3.31 ppm) δ 7.66 (s, 1H, 17-H), 7.00 (s, 1H, 19-H), 6.97 (s, 1H, 21-H), 6.51 (dd, *J* = 15.1, 10.8 Hz, 1H, 12-H), 6.01 (d, *J* = 10.8 Hz, 1H, 13-H), 5.42 (dd, *J* = 15.1, 7.0 Hz, 1H, 11-H), 5.36 (d, *J* = 8.3 Hz, 1H, 5-H), 4.32 (dd, *J* = 5.9, 5.9 Hz, 1H, 3-H), 3.45–3.40 (m, 1H, 10-H), 3.44–3.41 (m, 1H, 9-H), 3.37 (d, *J* = 14.4 Hz, 1H, 15-H_a), 3.30 (s, 3H, 10-OMe), 3.27 (d, *J* = 14.4 Hz, 1H, 15-H_b), 2.68 (d, *J* = 5.9 Hz, 2H, 2a & 2b), 2.38–2.28 (m, 1H, 6-H), 1.73 (s, 3H, 14-Me), 1.64 (s, 3H, 4-Me), 1.56–1.50 (m, 1H, 7-H_a), 1.53–1.48 (m, 1H, 8-H_a), 1.37–1.28 (m, 1H, 7-H_b), 1.28–1.20 (m, 1H, 8-H_b), 0.83 (d, *J* = 6.7 Hz, 3H, 6-Me) ppm; ^{13}C NMR

(125 MHz, methanol-d_4, methanol-d_4 = 49.00 ppm) δ 171.6 (s, C-1), 144.3 (s, C-16), 140.9 (s, C-18), 139.1 (s, C-14), 135.6 (s, C-4), 134.6 (s, C-20), 132.9 (d, C-5), 131.9 (d, C-12), 129.9 (d, C-11), 128.2 (d, C-13), 125.6 (d, C-21), 120.0 (d, C-17), 118.6 (d, C-19), 88.6 (d, C-10), 76.0 (d, C-9), 74.3 (d, C-3), 56.6 (q, 10-OMe), 46.1 (t, C-15), 42.6 (t, 2a & 2b), 34.6 (t, C-7), 33.3 (d, C-6), 31.6 (t, C-8), 21.3 (q, 6-Me), 16.9 (q, 14-Me), 13.3 (q, 4-Me) ppm; UPLC-MS [MeCN] t_R 2.18 min; HRMS-ESI (*m*/*z*): calcd for $C_{25}H_{34}ClNO_4Na$ $[M + Na]^+$ 470.2074; found: 470.2077.

7-Deoxy-9-hydro-9-*O*-carbamoyl-20-chloro-proansamitocin (11h)

^{1}H NMR (500 MHz, methanol-d_4, CHD_2OD = 3.31 ppm) δ 7.65 (s, 1H, 17-H), 7.02 (s, 1H, 19-H), 7.00 (s, 1H, 21-H), 6.56 (dd, *J* = 15.1, 10.8 Hz, 1H, 12-H), 6.04 (d, *J* = 10.8 Hz, 1H, 13-H), 5.51 (dd, *J* = 15.1, 7.3 Hz, 1H, 11-H), 5.35 (d, *J* = 8.2 Hz, 1H, 5-H), 4.71 (ddd, *J* = 7.2, 5.7, 5.5 Hz, 1H, 9-H), 4.37 (dd, *J* = 6.7, 5.8 Hz, 1H, 3-H), 3.71 (dd, *J* = 7.3, 7.2 Hz, 1H, 10-H), 3.36 (d, *J* = 16.0 Hz, 1H, 15-H_a), 3.31 (s, 3H, 10-OMe), 3.32 (d, *J* = 16.0 Hz, 1H, 15-H_b), 2.69 (d, *J* = 5.8 Hz, 1H, 2-H_a), 2.69 (d, *J* = 6.7 Hz, 1H, 2-H_b), 2.36–2.26 (m, 1H, 6-H), 1.74 (s, 3H, 14-Me), 1.66 (s, 3H, 4-Me), 1.64–1.54 (m, 1H, 8-H_a), 1.46–1.36 (m, 1H, 8-H_b), 1.41–1.32 (m, 2H, 7-H), 0.81 (d, *J* = 6.8 Hz, 3H, 6-Me) ppm; ^{13}C NMR (125 MHz, methanol-d_4, methanol-d_4 = 49.0 ppm) δ 171.5 (s, C-1), 160.0 (s, 9-$OCONH_2$), 144.2 (s, C-16), 140.9 (s, C-18), 139.3 (s, C-14), 135.8 (s, C-4), 134.6 (s, C-20), 133.1 (d, C-5), 131.3 (d, C-12), 129.3 (d, C-11), 127.9 (d, C-13), 125.8 (d, C-21), 120.3 (d, C-17), 118.9 (d, C-19), 85.3 (d, C-10), 77.4 (d, C-9), 74.6 (d, C-3), 56.9 (q, 10-OMe), 46.2 (t, C-15), 43.0 (t, C-2), 33.7 (t, C-7), 33.0 (d, C-6), 29.1 (t, C-8), 20.8 (q, 6-Me), 16.8 (q, 14-Me), 12.8 (q, 4-Me) ppm; UPLC-MS [MeCN] t_R 2.13 min; HRMS-ESI (*m*/*z*): $[M + Na]^+$ calcd for $C_{26}H_{35}ClN_2O_5Na$ 513.2132; found: 513.2132.

2.2 $\Delta^{10,12}$-Proansamitocin derivatives 12, 13a, 13b

All new metabolites listed in Table S2 and Table S3 were collected as colorless solids. By including the recovery of the starting compounds **9a** and **9b** overall 95% (9.2 µmol) and 77% (7.4 µmol) material, respectively, were (re)isolated.

Table S2: Chromatographic purification of proansamitocin-derivatives **12** and **13a/b**.

Sample	column	conditions	fractions
from **9a**: crude extract	CN-SP	H_2O:MeCN [ratio A:B], flow rate = 2.5 mL/min, gradient (*t* [min]/B [%]): (0/5) (5/5) (45/12) (80/28)	**9a** (t_R = 16.5 min)<br>F-1 (t_R = 30.0–35.0 min)
from **9b**: crude extract			F-1 (t_R = 16.0–31.0 min)<br>**13b** (t_R = 41.5 min)
**9a/F1**:	C18-SP	H_2O:MeOH [ratio A:B], flow rate = 2.25 mL/min gradient (*t* [min]/B [%]): (0/10) (10/10) (90/55)	**13a** (t_R = 87.5 min)
**9b/F1**:	C18-SP	H_2O:MeOH [ratio A:B], flow rate = 2.25 mL/min gradient (*t* [min]/B [%]): (0/10) (10/10) (90/45)	**9b** (t_R = 75.0 min)<br>**12** (t_R = 77.0 min)

Table S3: Isolated amounts.

Derivative	amount isolated	yield	
		fermentation	relation
**9a**	3.4 mg (7.4 µmol)	77%	81%
**13a**	0.8 mg (1.8 µmol)	18%	19%
**9b**	1.0 mg (2.2 µmol)	23%	30%
**13b**	1.6 mg (3.6 µmol)	37%	48%
**12**	0.8 mg (1.6 µmol)	17%	22%

7-Deoxy-14-hydroxy-$\Delta^{10,12}$-proansamitocin (13a, diastereomer 1)

^{1}H NMR (500 MHz, methanol-d_4, CHD_2OD = 3.31 ppm) δ 6.87 (dd, J = 1.9, 1.9 Hz, 1H, 17-H), 6.83 (d, J = 10.8 Hz, 1H, 11-H), 6.75 (dd, J = 1.9, 1.9 Hz, 1H, 19-H), 6.44 (dd, J = 1.9, 1.9 Hz, 1H, 21-H), 6.43 (dd, J = 15.6, 10.8 Hz, 1H, 12-H), 6.22 (d, J = 15.6 Hz, 1H, 13-H), 5.36 (dq, J = 9.9, 1.3 Hz, 1H, 5-H), 4.31 (dd, J = 9.7, 3.4 Hz, 1H, 3-H), 3.52 (s, 3H, 10-OMe), 2.82 (ddd, J = 16.5, 8.4, 6.6 Hz, 1H, 8-H_a), 2.80 (dd, J = 13.5, 3.4 Hz, 1H, 2-H_a), 2.75 (s, 2H, 15-H), 2.62 (ddd, J = 16.5, 6.5, 6.3 Hz, 1H, 8-H_b), 2.53 (dd, J = 13.5, 9.7 Hz, 1H, 2-H_b), 2.40 (dddq, J = 9.9, 9.1, 5.6, 6.4 Hz, 1H, 6-H), 1.82 (dddd, J = 13.5, 8.4, 6.5, 5.6 Hz, 1H, 7-H_a), 1.55 (d, J = 1.3 Hz, 3H, 4-Me), 1.38 (s, 3H, 14-Me), 1.37 (dddd, J = 13.5, 9.1, 6.6, 6.3 Hz, 1H, 7-H_b), 1.0 (d, J = 6.4 Hz, 3H, 6-Me) ppm; ^{13}C NMR (125 MHz, methanol-d_4, methanol-d_4 = 49.0 ppm) δ 200.0 (s, C-9), 171.0 (s, C-1), 158.4 (s, C-20), 152.3 (s, C-10), 149.6 (d, C-13), 140.2 (s, C-16), 139.8 (s, C-18), 138.4 (s, C-4), 131.0 (d, C-5), 130.5 (d, C-11), 121.5 (d, C-12), 115.9 (d, C-17), 114.6 (d, C-21), 105.9 (d, C-19), 74.4 (s, C-14), 73.2 (d, C-3), 60.7 (q, 10-OMe), 51.3 (t, C-15), 45.7 (t, C-2), 36.6 (t, C-8), 33.5 (t, C-7), 32.3 (d, C-6), 28.1 (q, 14-Me), 21.5 (q, 6-Me), 16.5 (q, 4-Me) ppm; UPLC-MS [MeOH] t_R 1.93 min; HRMS-ESI (m/z): $[M + Na]^+$ calcd for $C_{25}H_{33}NO_6Na$ 466.2206; found: 466.2199.

7-*O*-Carbamoyl-14-hydroxy-$\Delta^{10,12}$-proansamitocin (12)

^{1}H NMR (500 MHz, methanol-d_4, CHD_2OD = 3.31 ppm) δ 7.19 (dd, J = 1.9, 1.9 Hz, 1H, 19-H), 6.87 (d, J = 10.7 Hz, 1H, 11-H), 6.59 (dd, J = 1.9, 1.9 Hz, 1H, 17-H), 6.51 (dd, J = 15.5, 10.7 Hz, 1H, 12-H), 6.44 (dd, J = 1.9, 1.9 Hz, 1H, 21-H), 6.14 (d, J = 15.5 Hz, 1H, 13-H), 5.55 (ddq, J = 9.7, 1.3, 1.4 Hz, 1H, 5-H), 5.15 (ddd, J = 9.2, 5.1, 4.2 Hz, 1H, 7-H), 4.33 (dd, J = 10.2, 3.7 Hz, 1H, 3-H), 3.56 (s, 3H, 10-OMe), 3.05 (dd, J = 14.3, 5.1 Hz, 1H, 8-H_a), 2.88 (dd, J = 14.3, 9.2 Hz, 1H, 8-H_b), 2.76 (s, 2H, 15-H), 2.72 (dd, J = 3.7, 12.6 Hz, 1H, 2-H_a), 2.66 (ddq, J = 9.7, 4.2, 6.8 Hz, 1H, 6-H), 2.44 (dd, J = 12.6, 10.2 Hz, 1H, 2-H_b), 1.65 (d, J = 1.4 Hz, 3H, 4-Me), 1.35 (s, 3H, 14-Me), 1.05 (d, J = 6.8 Hz, 3H, 6-Me) ppm; ^{13}C NMR (125 MHz, methanol-d_4, methanol-d_4 = 49.0 ppm) δ 196.9 (s, C-9), 171.2 (s, C-1), 159.7 (s, 7-$OCONH_2$), 158.6 (s, C-20), 152.2 (s, C-10), 149.7 (d, C-13), 140.11 (s, C-16), 140.07 (s, C-18), 139.8 (s, C-4), 131.0 (d, C-11), 127.7 (d, C-5), 121.9 (d, C-12), 115.3 (d, C-17), 114.4 (d, C-21), 106.4 (d, C-19), 75.7 (d, C-7), 74.2 (s, C-14), 73.4 (d, C-3), 60.6 (q, 10-OMe), 51.7 (t, C-15), 47.2 (t, C-2), 43.2 (t, C-8), 37.0 (d, C-6), 26.7 (q, 14-Me), 16.6

(q, 4-Me), 14.6 (q, 6-Me) ppm; UPLC-MS [MeOH] t_R 1.68 min; HRMS-ESI (*m*/*z*): $[M + Na]^+$ calcd for $C_{26}H_{34}N_2O_8Na$ 525.2213; found: 525.2201.

7-Deoxy-14-hydroxy-$\Delta^{10,12}$-proansamitocin (13b, diastereomer 2)

^{1}H NMR (500 MHz, methanol-d_4, CHD_2OD = 3.31 ppm) δ 6.91 (dd, *J* = 1.9, 1.9 Hz, 1H, 17-H), 6.78 (d, *J* = 10.9 Hz, 1H, 11-H), 6.76 (dd, *J* = 1.9, 1.9 Hz, 1H, 19-H), 6.44 (dd, *J* = 15.6, 10.9 Hz, 1H, 12-H), 6.43 (dd, *J* = 1.9, 1.9 Hz, 1H, 21-H), 6.13 (d, *J* = 15.6 Hz, 1H, 13-H), 5.37 (dq, *J* = 9.5, 1.4 Hz, 1H, 5-H), 4.33 (dd, *J* = 9.7, 3.5 Hz, 1H, 3-H), 3.55 (s, 3H, 10-OMe), 2.81 (ddd, *J* = 16.5, 9.5, 5.2 Hz, 1H, 8-H_a), 2.80 (dd, *J* = 13.8, 3.5 Hz, 1H, 2-H_a), 2.753 (s, 1H, 15-H_a), 2.748 (s, 1H, 15-H_b), 2.60 (ddd, *J* = 16.5, 9.4, 6.7 Hz, 1H, 8-H_b), 2.56 (dd, *J* = 13.8, 9.7 Hz, 1H, 2-H_b), 2.43 (dddq, *J* = 9.5, 9.2, 5.4, 6.5 Hz, 1H, 6-H), 1.71 (dddd, *J* = 13.4, 9.5, 6.7, 5.4 Hz, 1H, 7-H_a), 1.64 (d, *J* = 1.4 Hz, 3H, 4-Me), 1.43 (dddd, *J* = 13.4, 9.4, 9.2, 5.2 Hz, 1H, 7-H_b), 1.35 (s, 3H, 14-Me), 1.0 (d, *J* = 6.5 Hz, 3H, 6-Me) ppm; ^{13}C NMR (125 MHz, methanol-d_4, methanol-d_4 = 49.0 ppm) δ 200.0 (s, C-9), 171.1 (s, C-1), 158.5 (s, C-20), 152.3 (s, C-10), 149.5 (d, C-13), 140.3 (s, C-16), 140.0 (s, C-18), 138.1 (s, C-4), 131.2 (d, C-5), 130.3 (d, C-11), 121.4 (d, C-12), 115.7 (d, C-17), 114.2 (d, C-21), 105.8 (d, C-19), 74.3 (s, C-14), 73.1 (d, C-3), 60.7 (q, 10-OMe), 51.7 (t, C-15), 45.4 (t, C-2), 37.4 (t, C-8), 33.9 (t, C-7), 32.7 (d, C-6), 27.0 (q, 14-Me), 21.4 (q, 6-Me), 16.5 (q, 4-Me) ppm; UPLC-MS [MeOH] t_R 1.99 min; HRMS-ESI (*m*/*z*): $[M + Na]^+$ calcd for $C_{25}H_{33}NO_6Na$ 466.2206; found: 466.2209.

2.3 20-O-Methyl-proansamitocin derivatives 14–16

The derivatives **14** (23.7 mg, 44.3 µmol, 24.7 mg/L), **15** (2.8 mg, 5.6 µmol, 2.9 mg/L) and **16** (0.7 mg, 1.6 µmol, 0.7 mg/L) were obtained as colorless solids following extraction and purification (Table S4) from a total fermentation volume of 960 mL. No signals could be assigned to the quaternary carbons C-18 and C-19 of compound **14** [S5] and C-18 of compound **15**, indicated by n.d. = not determined.

Table S4: Chromatographic purification of proansamitocin-derivatives **14-16**.

Sample	column	conditions	fractions
crude extract	SiO_2	petroleum ether:ethyl acetate 4:1 → ethyl acetate	F-1 (R_f (EE) 0.4–0.05)
F-1	Sephadex LH 20	MeOH	F-2 (M_R = 400–700 Da)
F-2	C18-$P_{[B]}$	H_2O[+ 0.1% FA]:MeOH[+ 0.1% FA] [A:B],<br>flow rate = 15 mL/min<br>gradient (*t* [min]/B [%]):<br>(0/20) (5/20) (90/100)	F-3 (t_R = 62.0–64.0 min)<br>F-4 (t_R = 64.0–65.0 min)<br>F-5 (t_R = 65.0–66.0 min)
F-3	CN-SP	H_2O:MeCN [A:B],<br>flow rate = 2.5 mL/min<br>gradient (*t* [min]/B [%]):<br>(0/5) (5/5) (45/18) (80/35) (90/50)	**14** (t_R = 76.5 min)
F-4	CN-SP	H_2O:MeCN [A:B],<br>flow rate = 2.5 mL/min<br>gradient (*t* [min]/B [%]):<br>(0/5) (5/5) (45/18) (80/35) (90/50)	**16** (t_R = 69.5 min)<br>**15** (t_R = 74.0 min)<br>**14** (t_R = 77.5 min)
F-5	CN-SP	H_2O:MeCN [A:B],<br>flow rate = 2.5 mL/min<br>gradient (*t* [min]/B [%]):<br>(0/5) (5/5) (45/18) (80/35) (90/50)	**15** (t_R = 73.0 min)

7-*O*-Carbamoyl-20-*O*-methyl-proansamitocin (14)

^1H NMR (400 MHz, methanol-d_4, CHD_2OD = 3.31 ppm) δ 7.60 (s, 1H, 17-H), 6.63 (dd, *J* = 15.6, 10.7 Hz, 1H, 12-H), 6.54 (s, 1H, 21-H), 6.45 (s, 1H, 19-H), 6.10 (d, *J* = 10.7 Hz, 1H, 13-H), 5.57 (dd, *J* = 15.6, 9.2 Hz, 1H, 11-H), 5.46 (d, *J* = 9.0 Hz, 1H, 5-

H), 4.29 (bdd, J = 11.6, 10.4 Hz, 1H, 7-H), 4.22 (bd, J = 6.5 Hz, 1H, 3-H), 3.77 (s, 3H, 20-OMe), 3.56 (d, J = 9.2 Hz, 1H, 10-H), 3.41 (d, J = 13.8 Hz, 1H, 15-H_a), 3.34 (s, 3H, 10-OMe), 3.15 (d, J = 13.8 Hz, 1H, 15-H_b), 2.81 (dd, J = 15.7, 2.7 Hz, 1H, 2-H_a), 2.72 (dd, J = 15.7, 6.5 Hz, 1H, 2-H_b), 2.70–2.59 (m, 1H, 6-H), 1.96 (bd, J = 14.1 Hz, 1H, 8-H_a), 1.68 (s, 3H, 14-Me), 1.65 (s, 3H, 4-Me), 1.43 (dd, J = 14.1, 11.6 Hz, 1H, 8-H_b), 1.14 (d, J = 6.5 Hz, 3H, 6-Me) ppm; ^{13}C NMR (100 MHz, methanol-d_4, methanol-d_4 = 49.00 ppm) δ 171.6 (s, C-1), 161.3 (s, C-20), 156.0 (s, 7-OCONH), 143.5 (s, C-16), 140.9 (s, C-14), n.d. (s, C-18), 139.1 (s, C-4), 135.0 (d, C-12), 127.9 (d, C-13), 126.7 (d, C-11), 125.8 (d, C-5), 113.1 (d, C-17), 111.8 (d, C-21), 103.3 (d, C-19), 89.2 (d, C-10), 82.5 (s, C-9), 79.7 (d, C-7), 73.3 (d, C-3), 56.4 (q, 10-OMe), 55.7 (q, 20-OMe), 46.8 (t, C-15), 41.0 (t, C-2), 38.4 (d, C-6), 36.2 (t, C-8), 18.0 (q, 6-Me), 16.7 (q, 14-Me), 15.0 (q, 4-Me) ppm; UPLC-MS [MeOH] t_R 2.39 min; HRMS-ESI (m/z): $[M + Na]^+$ calcd for $C_{27}H_{36}N_2O_7Na$ 523.2420; found: 523.2429.

_N_-Desmethyl-4,5-desepoxy-maytansinol (15)

^{1}H NMR (400 MHz, methanol-d_4, CHD_2OD = 3.31 ppm) δ 7.00–6.68 (m, 2H, 2 * Ar-H), 6.63 (bdd, J = 14.8, 10.8 Hz, 1H, 12-H), 6.10–5.84 (m, 1H, 13-H), 5.59–5.45 (m, 1H, 11-H), 5.48–5.35 (m, 1H, 5-H), 4.36–4.26 (m, 1H, 7-H), 4.28–4.21 (m, 1H, 3-H), 3.90 (bs, 3H, 20-OMe), 3.57 (d, J = 9.2 Hz, 1H, 10-H), 3.44 (d, J = 14.3 Hz, 1H, 15-H_a), 3.34 (s, 3H, 10-OMe), 3.25 (d, J = 14.3 Hz, 1H, 15-H_b), 3.06–2.65 (m, 2H, 2-H), 2.69–2.55 (m, 1H, 6-H), 1.76 (bs, 3H, 14-Me), 1.73–1.63 (m, 3H, 4-Me), 1.48–1.30 (m, 2H, 8-H), 1.13 (d, J = 6.5 Hz, 3H, 6-Me) ppm; ^{13}C NMR (100 MHz, methanol-d_4, methanol-d_4 = 49.00 ppm) δ 171.4 (s, C-1), 156.6 (s, C-20), 155.9 (s, 7-OCONH), 141.2 (s, C-16), 139.2 (s, C-14), n.d. (s, C-18), 137.0 (s, C-4), 134.5 (d, C-12), 128.5 (d, C-13), 127.2 (d, C-11), 125.1 (d, C-5), n.d. (s, C-19), 115.7 (d, C-17), 110.1 (d, C-21), 89.0 (d, C-10), 82.3 (s, C-9), 79.6 (d, C-7), 72.9 (d, C-3), 56.9 (q, 20-OMe), 56.6 (q, 10-OMe), 46.4 (t, C-15), 40.9 (t, C-2), 38.5 (d, C-6), 36.2 (t, C-8), 17.9 (q, 6-Me), 17.0 (q, 14-Me), 15.0 (q, 4-Me) ppm; UPLC-MS [MeOH] t_R 2.37 min; HRMS-ESI (m/z): $[M + H]^+$ calcd for $C_{27}H_{36}ClN_2O_7$ 535.2211; found: 535.2218.

7-Desoxy-20-_O_-methyl-proansamitocin (16)

^{1}H NMR (500 MHz, methanol-d_4, CHD_2OD = 3.31 ppm) δ 6.93 (dd, J = 1.9, 1.9 Hz, 1H, 17-H), 6.81 (ddd, J = 15.2, 10.9, 0.8 Hz, 1H, 12-H), 6.70 (dd, J = 1.9, 1.9 Hz, 1H, 19-H), 6.56 (dd, J = 1.9, 1.9 Hz, 1H, 21-H), 6.05 (d, J = 10.9 Hz, 1H, 13-H), 5.34 (dd,

J = 15.2, 7.9 Hz, 1H, 11-H), 5.09 (dq, *J* = 9.1, 1.1 Hz, 1H, 5-H), 4.47 (d, *J* = 7.9 Hz, 1H, 10-H), 4.33 (dd, *J* = 9.9, 5.1 Hz, 1H, 3-H), 3.77 (s, 3H, 20-OMe), 3.33 (d, *J* = 13.3 Hz, 1H, 15-H_a), 3.32 (s, 3H, 10-OMe), 3.17 (d, *J* = 13.3 Hz, 1H, 15-H_b), 2.60 (dd, *J* = 12.6, 5.1 Hz, 1H, 2-H_a), 2.58 (ddd, *J* = 15.9, 9.8, 5.7 Hz, 1H, 8-H_a), 2.56 (dd, *J* = 12.6, 9.9 Hz, 1H, 2-H_b), 2.31 (ddd, *J* = 15.9, 5.9, 5.6 Hz, 1H, 8-H_b), 1.98 (dddq, *J* = 10.2, 9.1, 3.6, 6.5 Hz, 1H, 6-H), 1.82 (dddd, *J* = 13.6, 9.8, 5.9, 3.6 Hz, 1H, 7-H_a), 1.71 (d, *J* = 1.1 Hz, 3H, 14-Me), 1.63 (d, *J* = 1.1 Hz, 3H, 4-Me), 1.17 (dddd, *J* = 13.6, 10.2, 5.7, 5.6 Hz, 1H, 7-H_b), 0.49 (d, *J* = 6.5 Hz, 3H, 6-Me) ppm; ^{13}C NMR (125 MHz, methanol-d_4, methanol-d_4 = 49.00 ppm) δ 210.1 (s, C-9), 171.2 (s, C-1), 161.2 (s, C-20), 143.0 (s, C-16), 142.2 (s, C-14), 140.2 (s, C-18), 135.3 (s, C-4), 134.4 (d, C-5), 133.7 (d, C-12), 126.5 (d, C-13), 126.0 (d, C-11), 114.5 (d, C-17), 112.5 (d, C-21), 105.7 (d, C-19), 89.1 (d, C-10), 76.2 (d, C-3), 56.9 (q, 10-OMe), 55.7 (q, 20-OMe), 47.1 (t, C-15), 44.5 (t, C-2), 36.8 (t, C-8), 32.6 (t, C-7), 32.0 (d, C-6), 19.5 (q, 6-Me), 16.7 (q, 14-Me), 11.3 (q, 4-Me) ppm; UPLC-MS [MeOH] t_R 2.45 min; HRMS-ESI (*m*/*z*): [M + Na]$^+$ calcd for $C_{26}H_{35}NO_5Na$ 464.2413; found: 464.2404.

3. Cell proliferation assay

Cell lines were obtained from DMSZ (U-937 ACC 5; A-431 ACC 91) or ATCC (SK-OV-3 HTB-77; PC-3 CRL-1435). Growth inhibition was measured in microtiter plates. 60 µL of serial dilutions of the test compounds were added to 120 µL aliquots of a cell suspension (50.000/mL) in 96-well plates and incubated at 37 °C and 10% CO_2 for 5 days. MTT [3(4,5-dimethylthiazol-2-yl)2,5-diphenyltetrazolium bromide] was used to measure growth and viability of the cells, which are capable of reducing it to a violet formazan product. 20 µL MTT in phosphate buffered saline (PBS) were added to a final concentration of 0.5 mg/mL. After 2 h the precipitate of formazan crystals was centrifuged, and the supernatant discarded. The precipitate was washed with PBS (100 µL) and dissolved in isopropanol (100 µL) containing 0.4% hydrochloric acid. The microplates were measured at 595 nm using an ELISA plate reader. All experiments were carried out as duplicate parallel experiments. The percentage of viable cells was calculated as the mean with respect to the controls, which were set to 100%. In the case of U-937 the WST-1 assay from Roche was employed.

4. NMR spectra

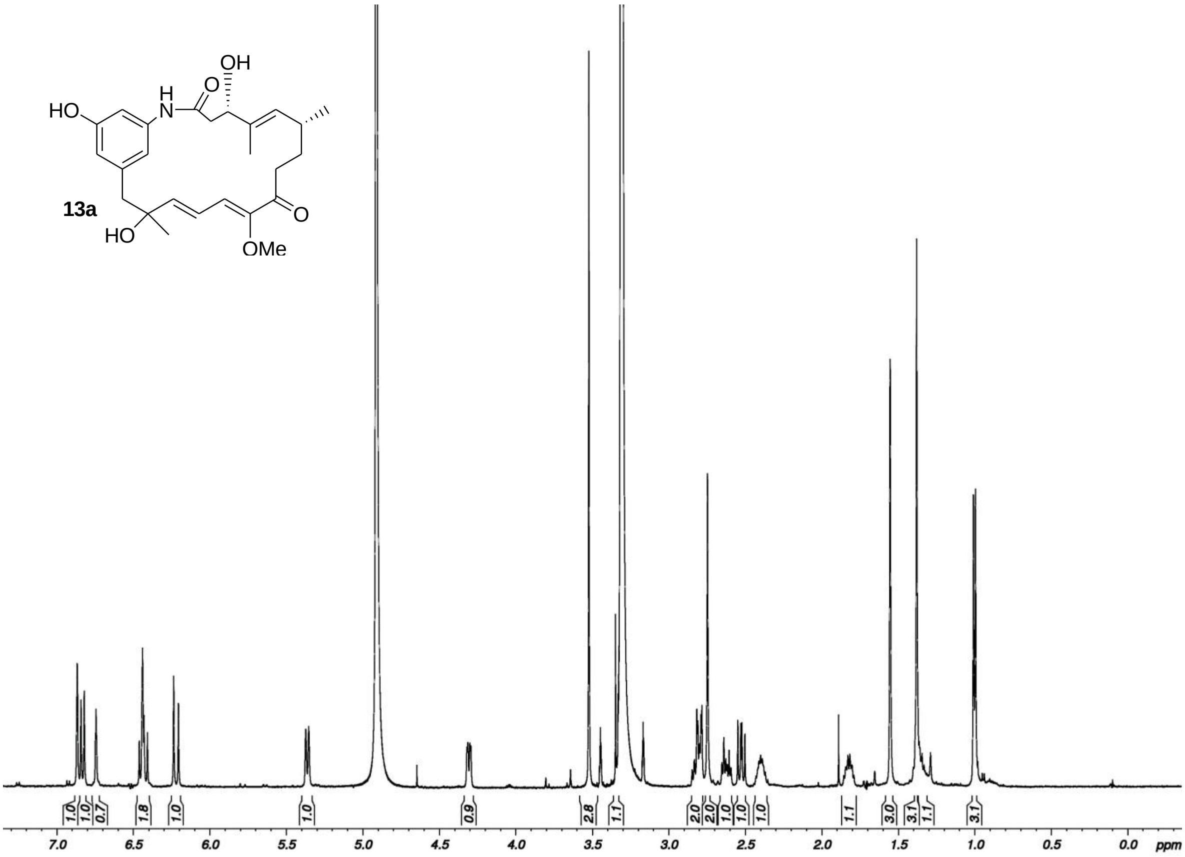

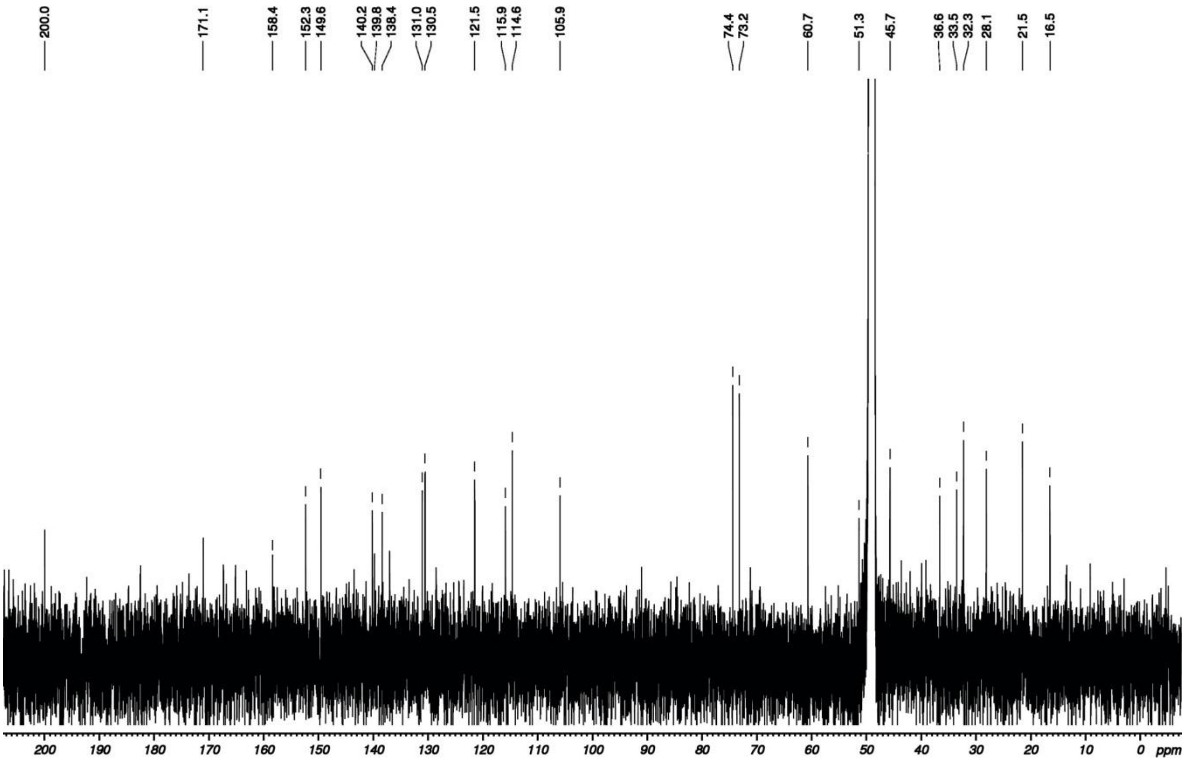

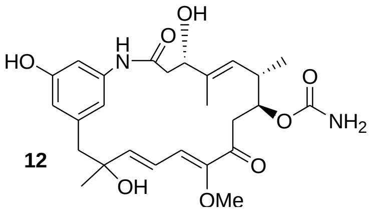
OH
H
N
O
HO
O
NH2
O
O
OH
OMe

12

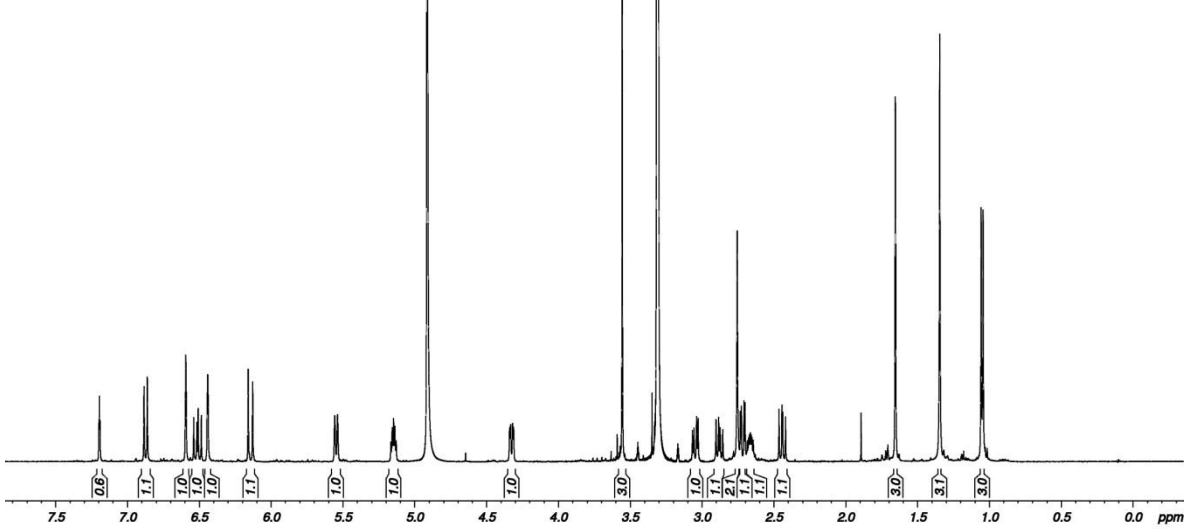
0.6
1.1
1.0
1.0
1.0
1.1
1.0
1.0
1.0
3.0
1.0
1.1
2.1
1.1
1.1
1.1
3.0
3.1
3.0
7.5 7.0 6.5 6.0 5.5 5.0 4.5 4.0 3.5 3.0 2.5 2.0 1.5 1.0 0.5 0.0 ppm

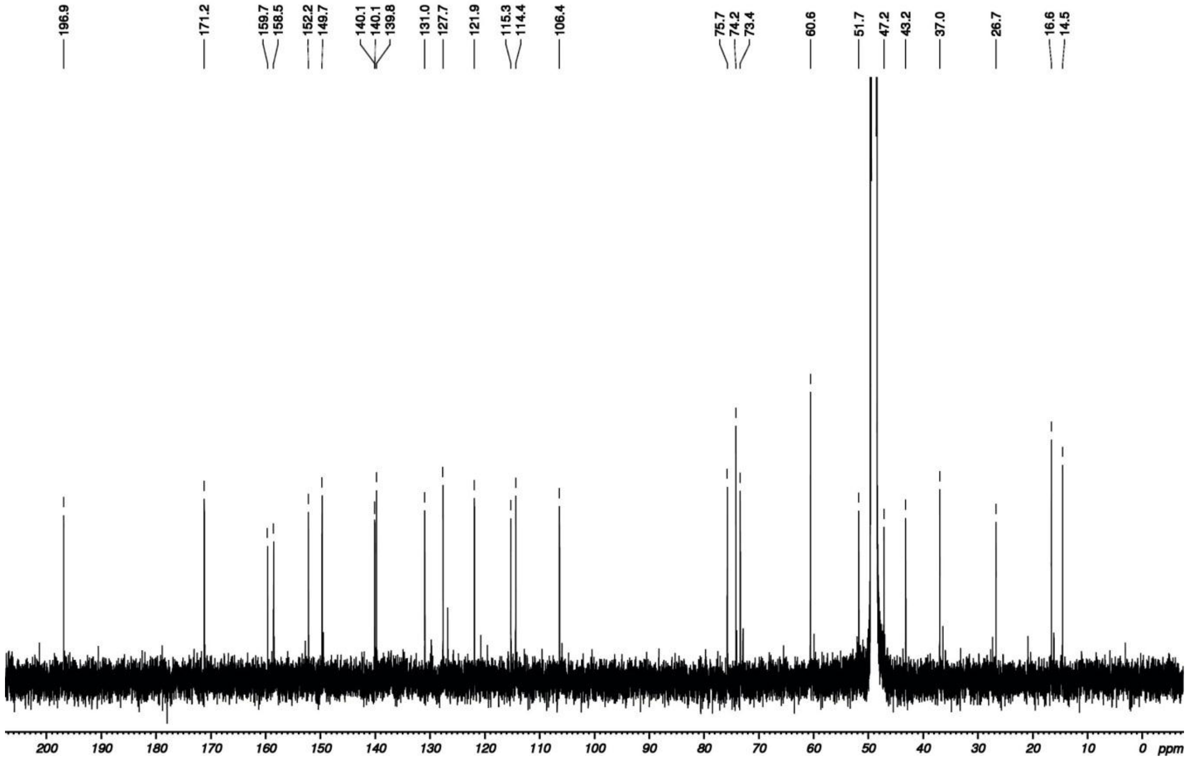
196.9
171.2
159.7
158.5
152.2
149.7
140.1
140.1
139.8
131.0
127.7
121.9
115.3
114.4
106.4
75.7
74.2
73.4
60.6
51.7
47.2
43.2
37.0
26.7
16.6
14.5
200 190 180 170 160 150 140 130 120 110 100 90 80 70 60 50 40 30 20 10 0 ppm

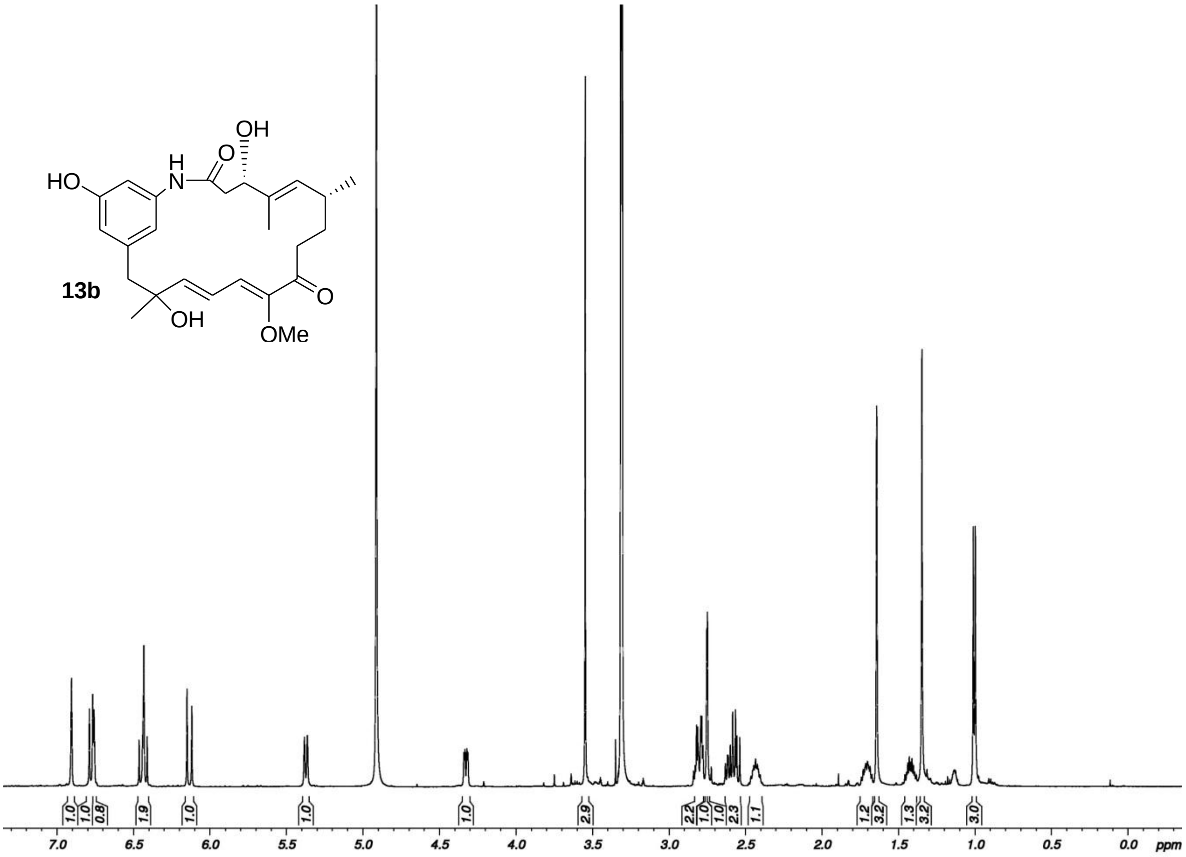
OH
H
HO
N
O
13b
OH
O
OMe
1.0 1.0 0.8 1.9 1.0 1.0 1.0 2.9 2.2 1.0 1.0 2.3 1.1 1.2 3.2 1.3 3.2 3.0
7.0 6.5 6.0 5.5 5.0 4.5 4.0 3.5 3.0 2.5 2.0 1.5 1.0 0.5 0.0 ppm

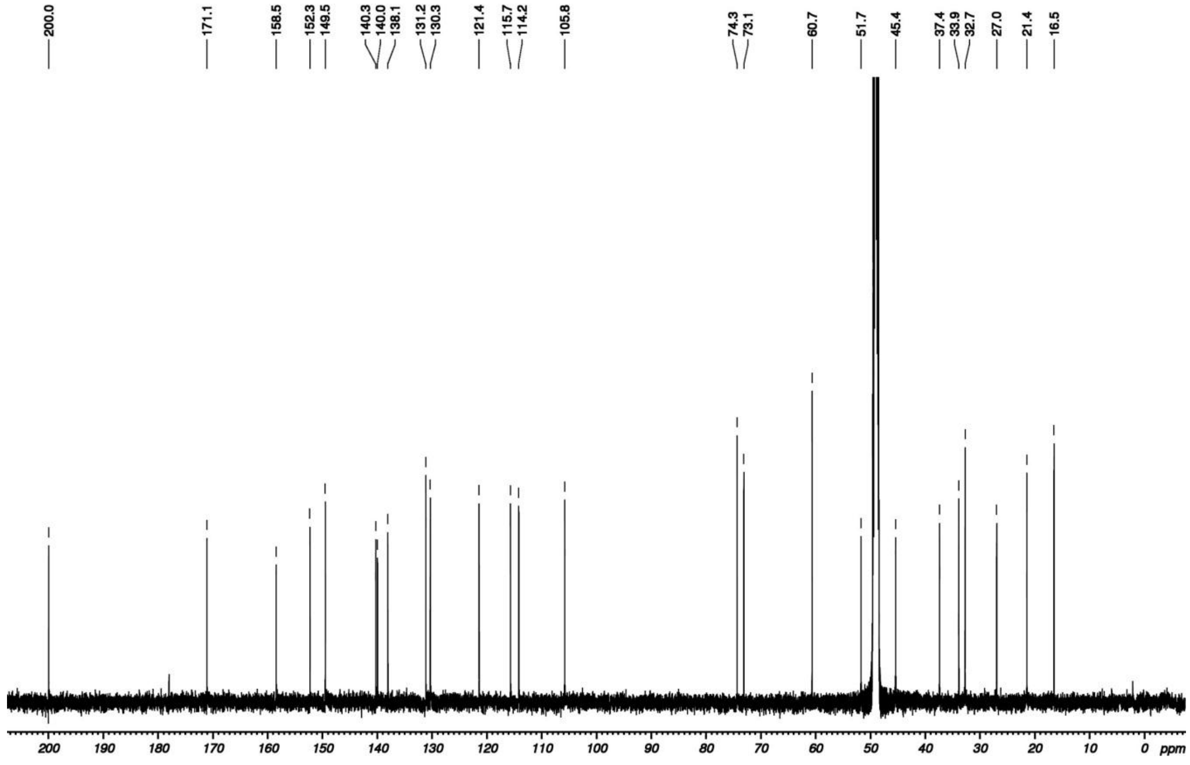
200.0
171.1
158.5
152.3
149.5
140.3
140.0
138.1
131.2
130.3
121.4
115.7
114.2
105.8
74.3
73.1
60.7
51.7
45.4
37.4
33.9
32.7
27.0
21.4
16.5
200 190 180 170 160 150 140 130 120 110 100 90 80 70 60 50 40 30 20 10 0 ppm

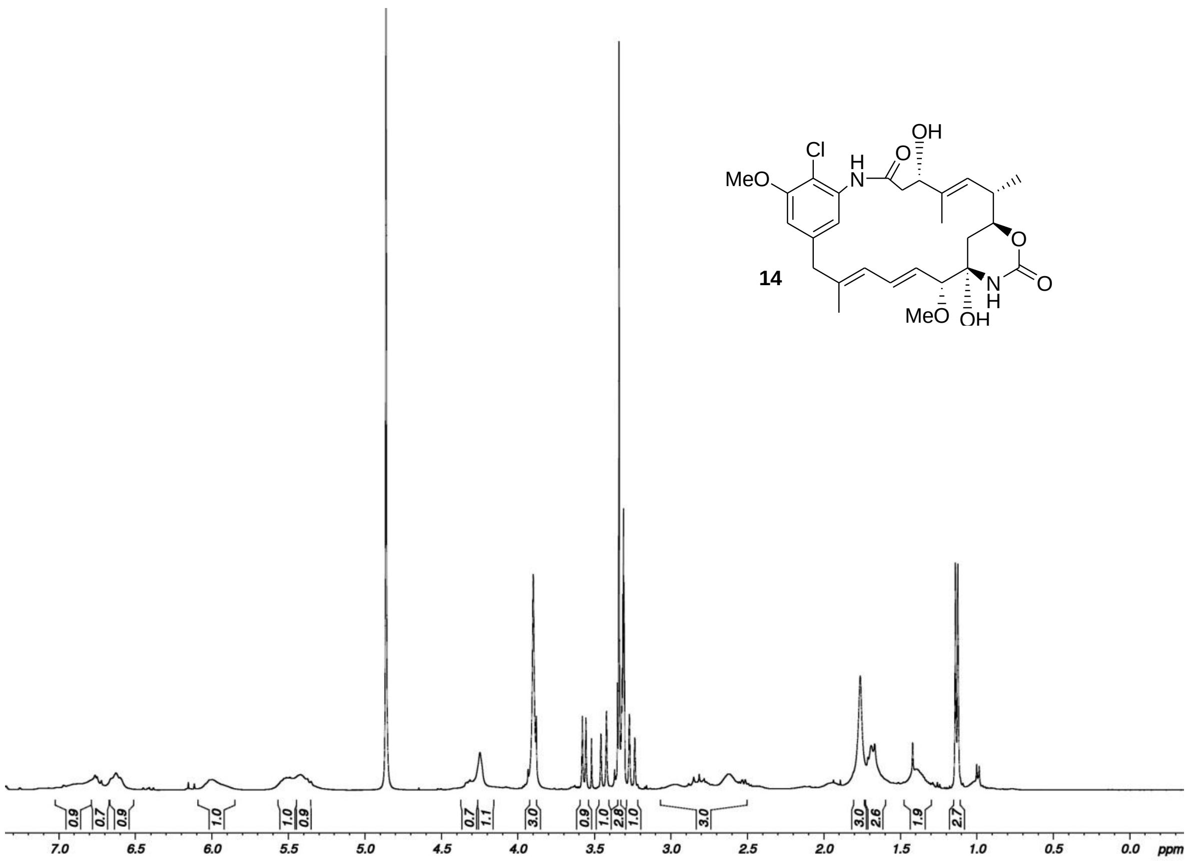

Cl
OH
MeO
H
N
O
O
O
14
N
H
O
MeO
OH
0.9 0.7 0.9 1.0 1.0 0.9 0.7 1.1 3.0 0.9 1.0 2.8 1.0 3.0 3.0 2.6 1.9 2.7
7.0 6.5 6.0 5.5 5.0 4.5 4.0 3.5 3.0 2.5 2.0 1.5 1.0 0.5 0.0 ppm

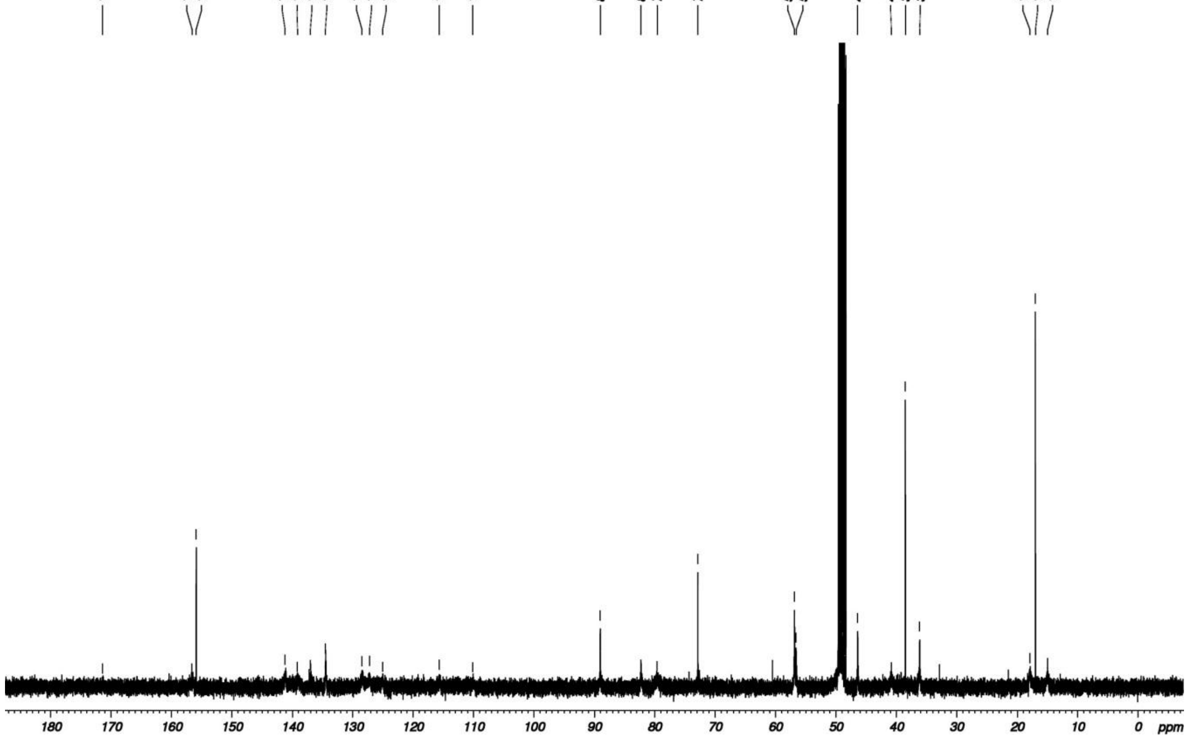

171.4
156.6 155.9
141.2 139.2 137.0 134.5
128.5 127.2 125.1
115.7
110.1
89.0
82.3 79.6
72.9
56.9 56.6
46.4
40.9 38.5 36.2
17.9 17.0 15.0
180 170 160 150 140 130 120 110 100 90 80 70 60 50 40 30 20 10 0 ppm

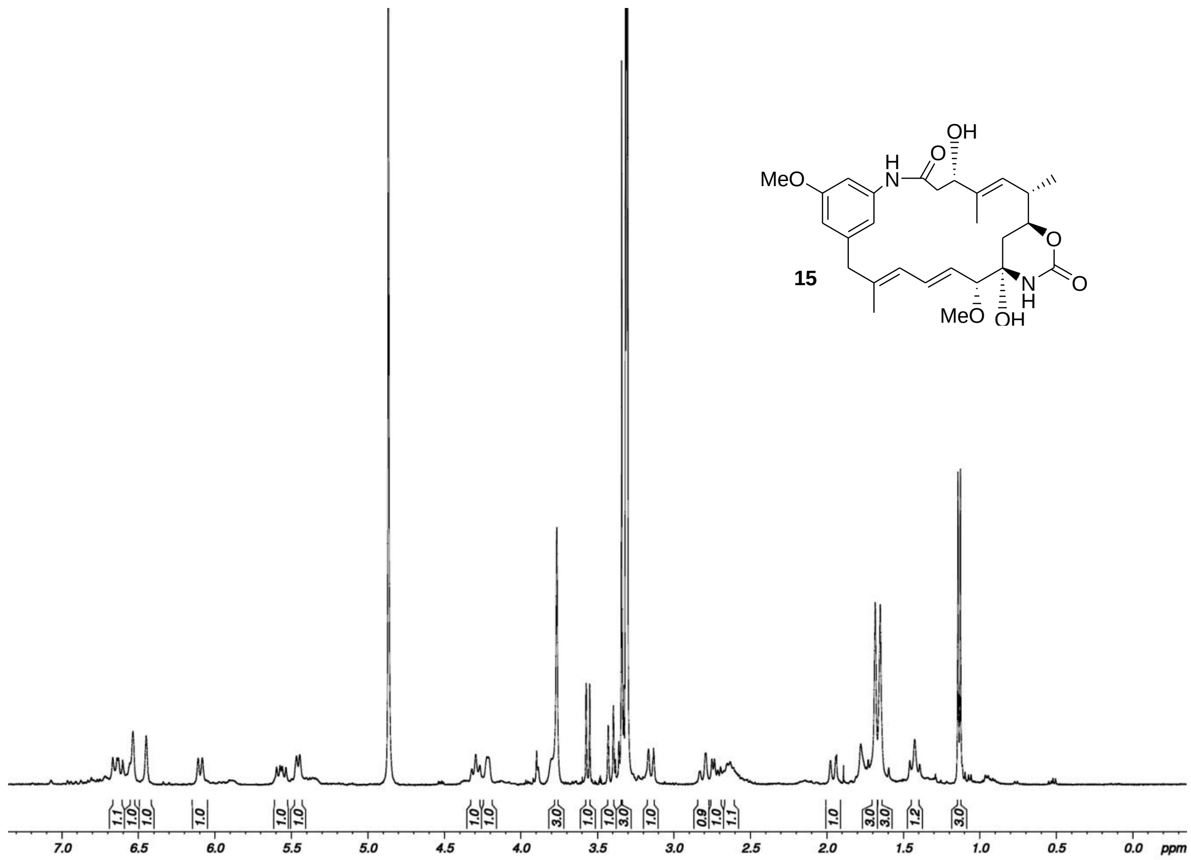
OH
H
N
O
MeO
O
N
H
O
MeO
OH
15
1.1
1.0
1.0
1.0
1.0
1.0
1.0
1.0
3.0
1.0
1.0
3.0
1.0
0.9
1.0
1.1
1.0
3.0
3.0
1.2
3.0
7.0
6.5
6.0
5.5
5.0
4.5
4.0
3.5
3.0
2.5
2.0
1.5
1.0
0.5
0.0
ppm

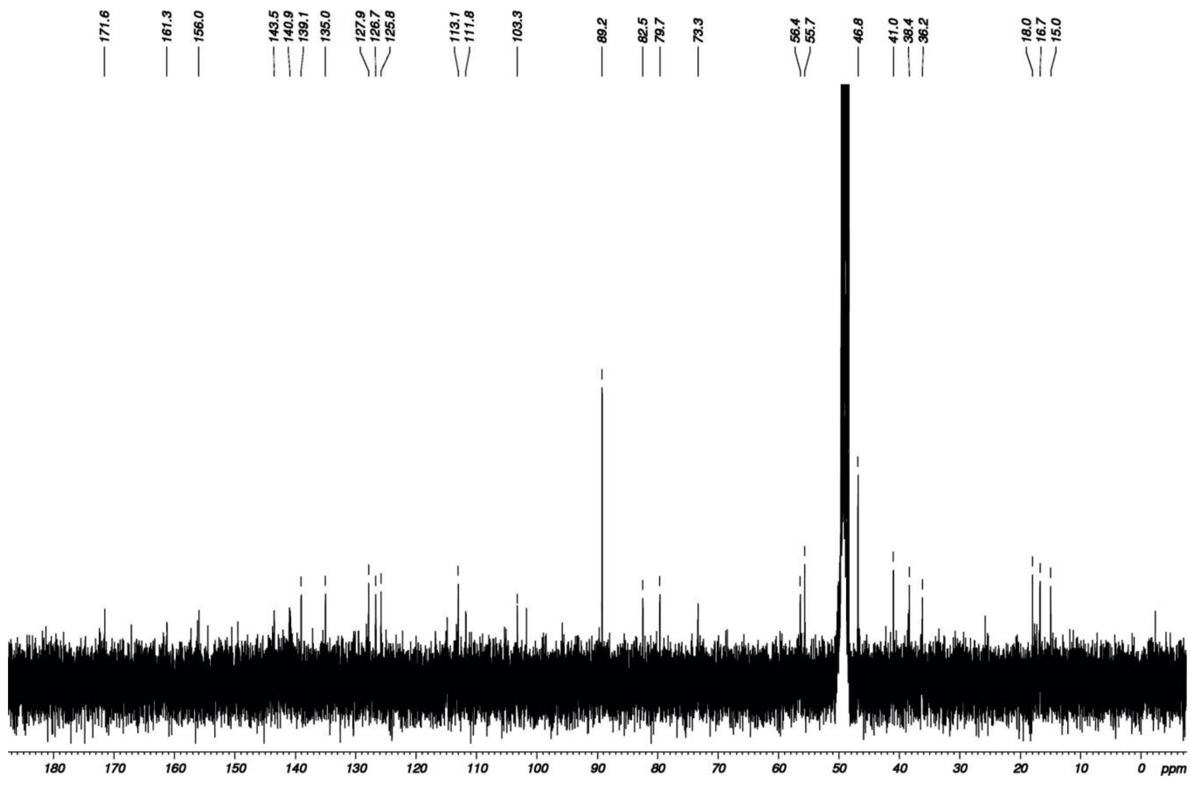
171.6
161.3
156.0
143.5
140.9
139.1
135.0
127.9
126.7
125.8
113.1
111.8
103.3
89.2
82.5
79.7
73.3
56.4
55.7
46.8
41.0
38.4
36.2
18.0
16.7
15.0
180
170
160
150
140
130
120
110
100
90
80
70
60
50
40
30
20
10
0
ppm

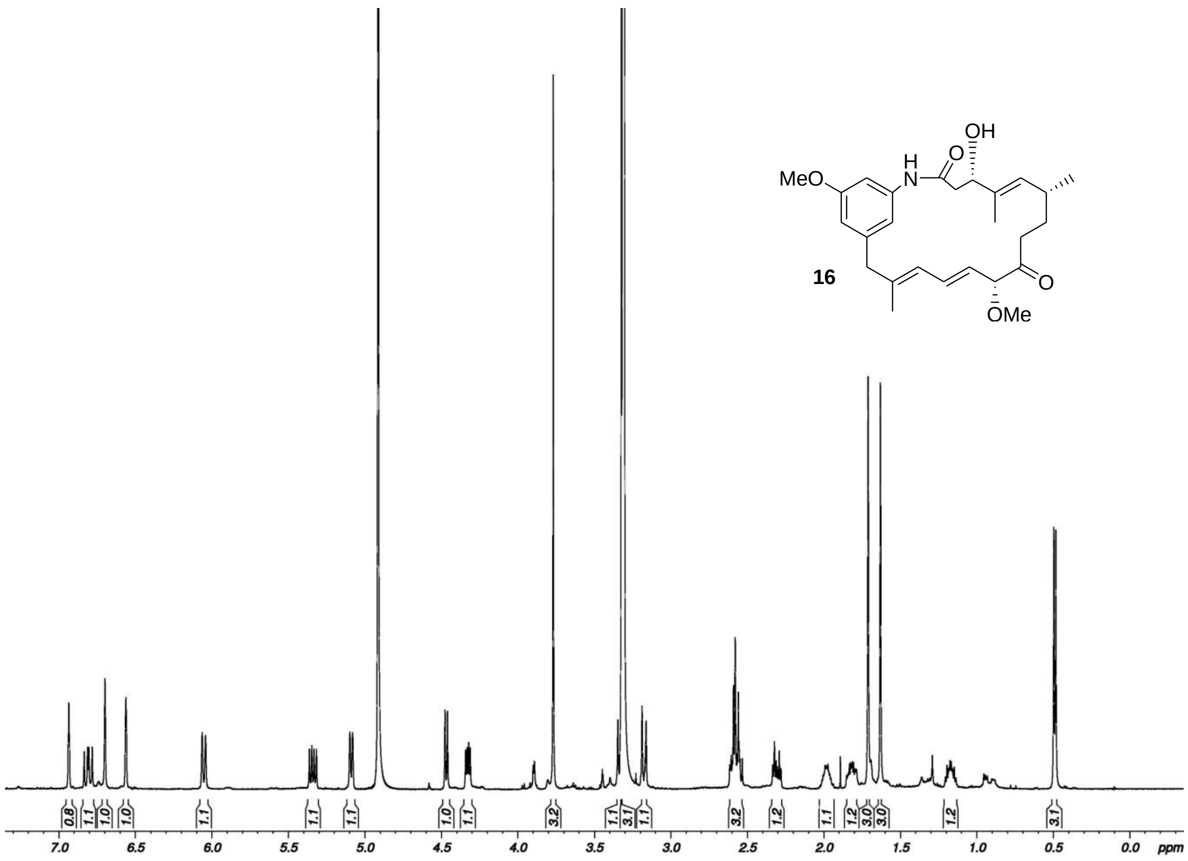
OH
H
N
O
MeO
16
O
OMe
0.8 1.1 1.0 1.0 1.1 1.1 1.1 1.0 1.1 3.2 1.1 3.1 1.1 3.2 1.2 1.1 1.2 3.0 3.0 1.2 3.1
7.0 6.5 6.0 5.5 5.0 4.5 4.0 3.5 3.0 2.5 2.0 1.5 1.0 0.5 0.0 ppm

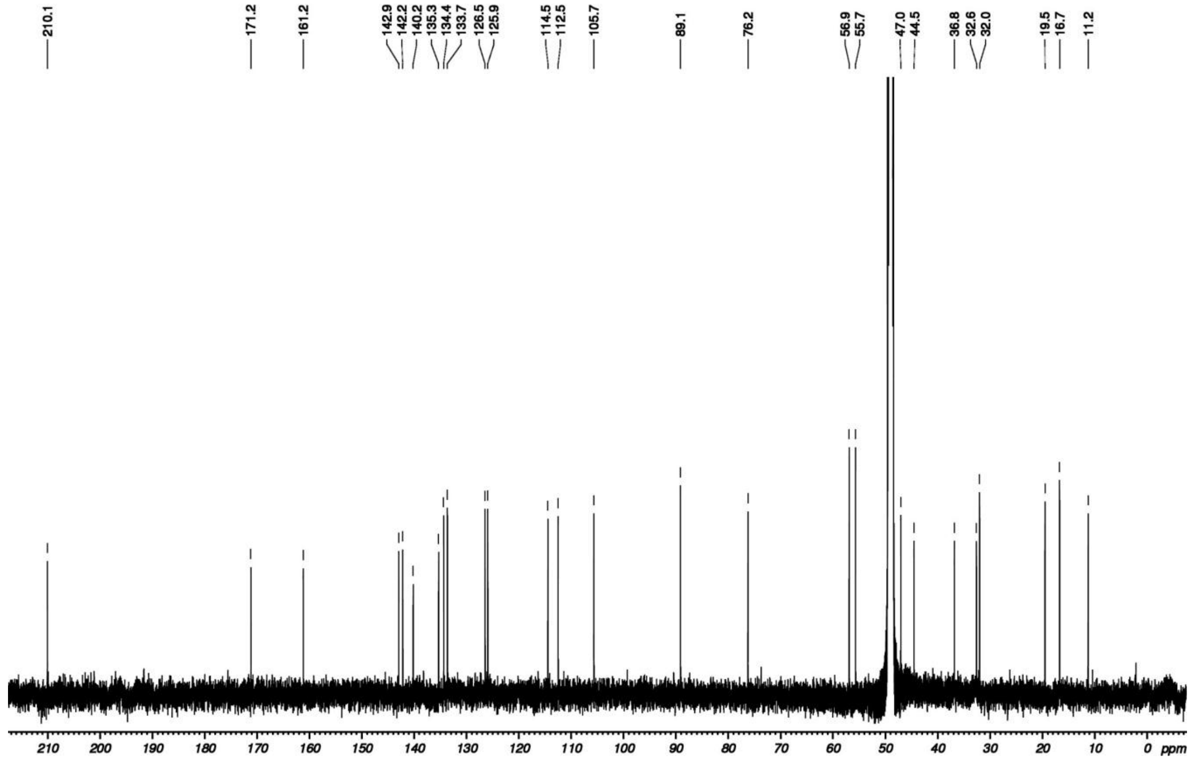
210.1
171.2
161.2
142.9 142.2 140.2 135.3 134.4 133.7 126.5 125.9
114.5 112.5
105.7
89.1
76.2
56.9 55.7
47.0 44.5
36.8 32.6 32.0
19.5 16.7 11.2
210 200 190 180 170 160 150 140 130 120 110 100 90 80 70 60 50 40 30 20 10 0 ppm

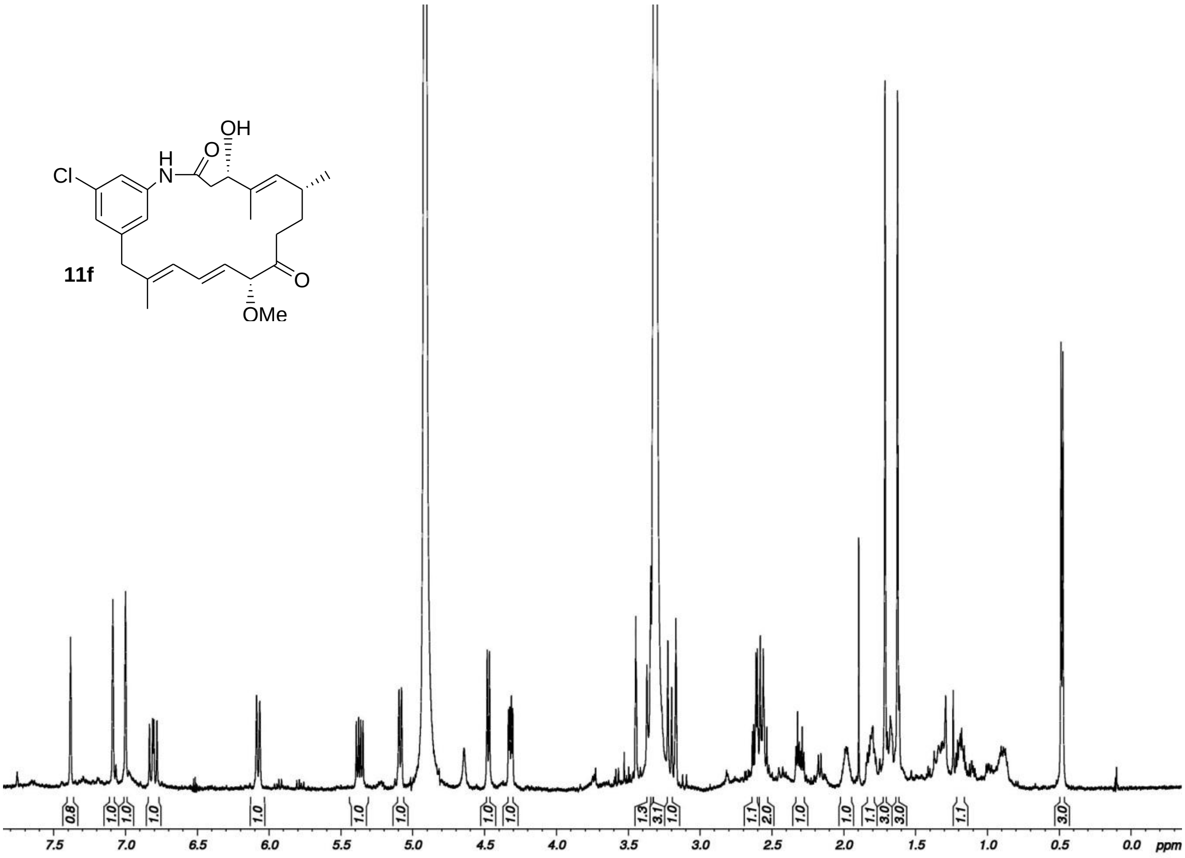
OH
H
N
O
Cl
O
OMe
0.8 1.0 1.0 1.0 1.0 1.0 1.0 1.0 1.0 1.3 3.1 1.0 1.1 2.0 1.0 1.0 1.1 3.0 3.0 1.1 3.0
7.5 7.0 6.5 6.0 5.5 5.0 4.5 4.0 3.5 3.0 2.5 2.0 1.5 1.0 0.5 0.0 ppm

11f

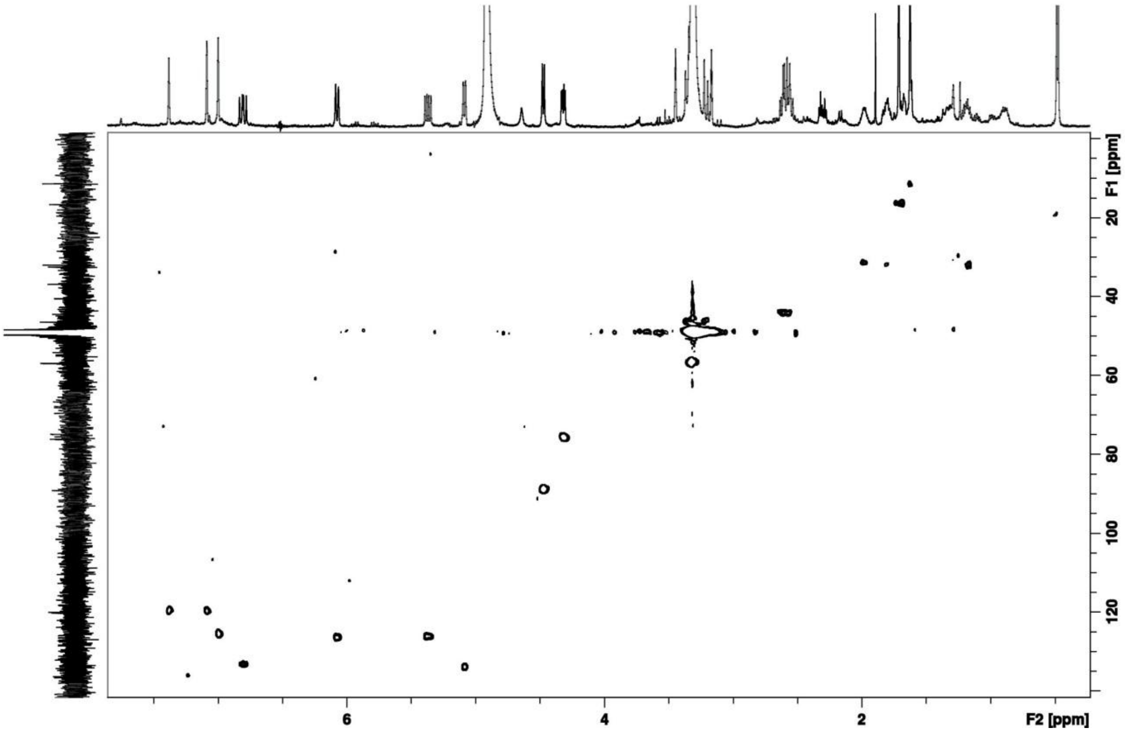
F1 [ppm]
20
40
60
80
100
120
6
4
2
F2 [ppm]

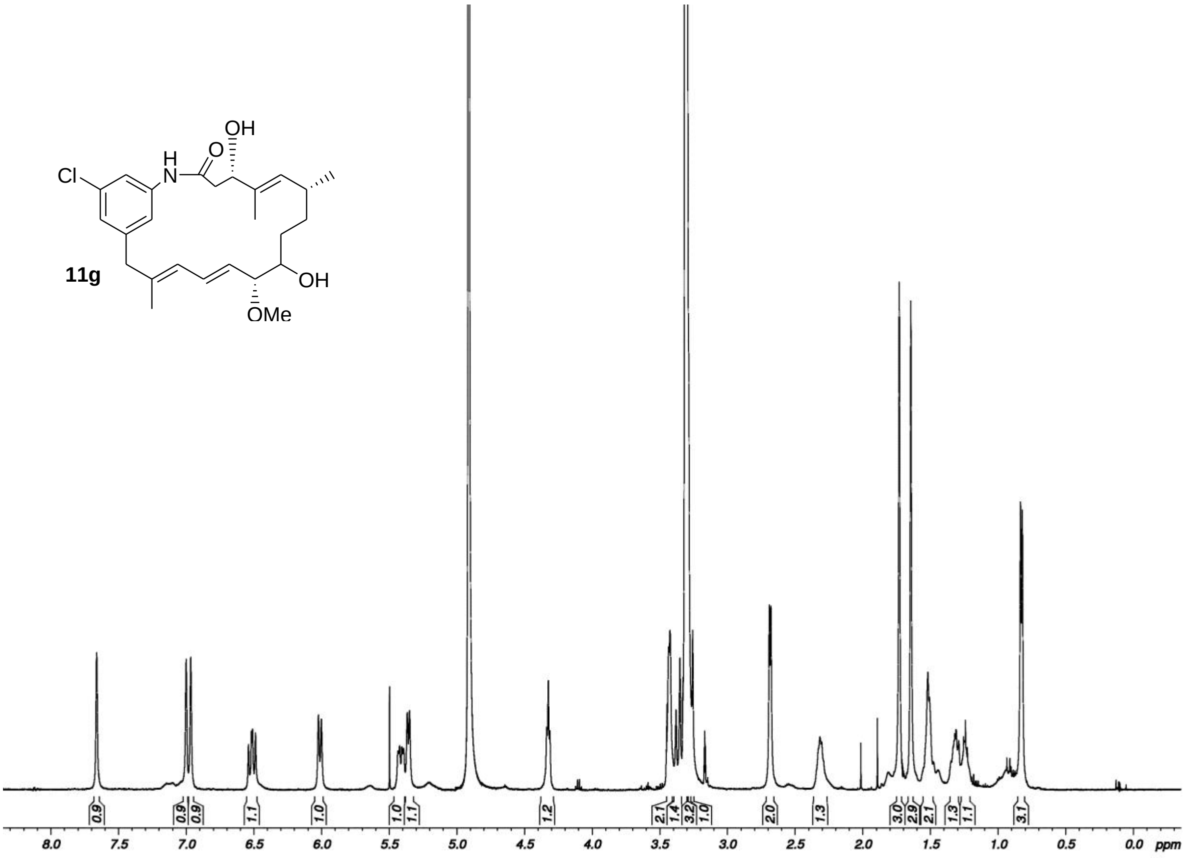
Cl
H
N
O
OH
OH
OMe
11g
0.9
0.9
0.9
1.1
1.0
1.0
1.1
1.2
2.1
1.4
3.2
1.0
2.0
1.3
3.0
2.9
2.1
1.3
1.1
3.1
8.0 7.5 7.0 6.5 6.0 5.5 5.0 4.5 4.0 3.5 3.0 2.5 2.0 1.5 1.0 0.5 0.0 ppm

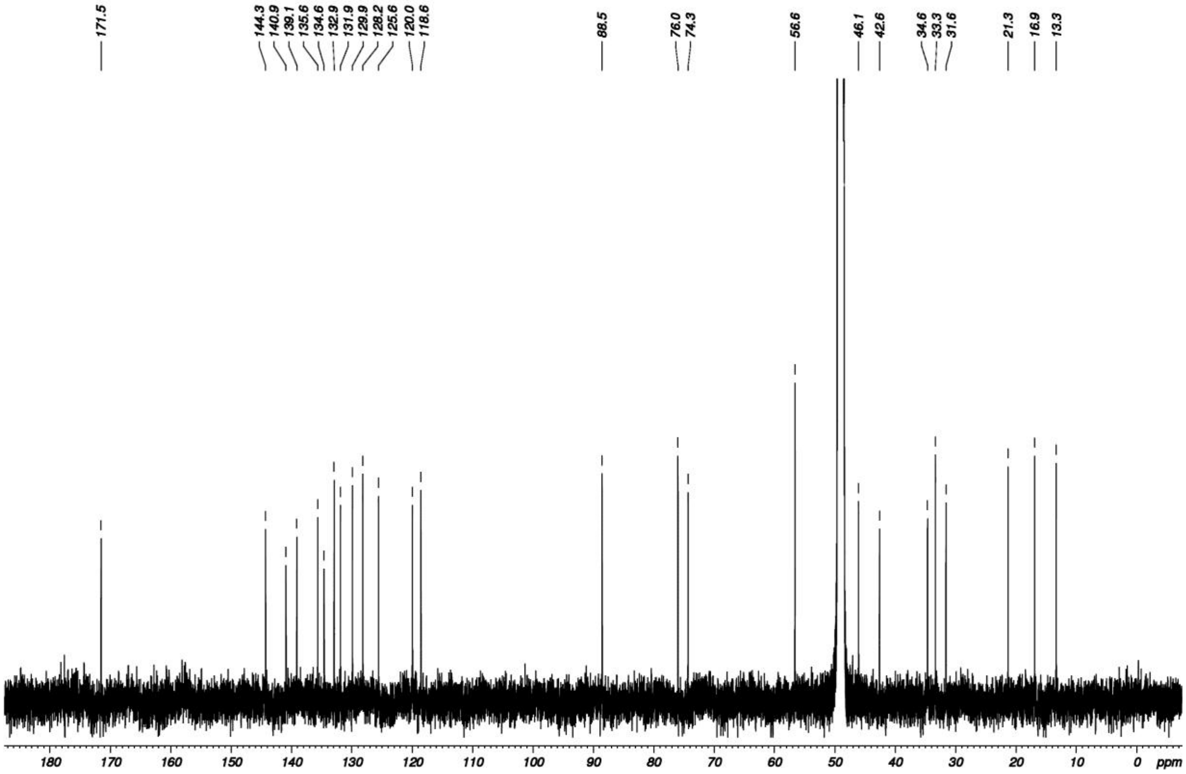
171.5
144.3
140.9
139.1
135.6
134.6
132.9
131.9
129.9
128.2
125.6
120.0
118.6
88.5
76.0
74.3
56.6
46.1
42.6
34.6
33.3
31.6
21.3
16.9
13.3
180 170 160 150 140 130 120 110 100 90 80 70 60 50 40 30 20 10 0 ppm

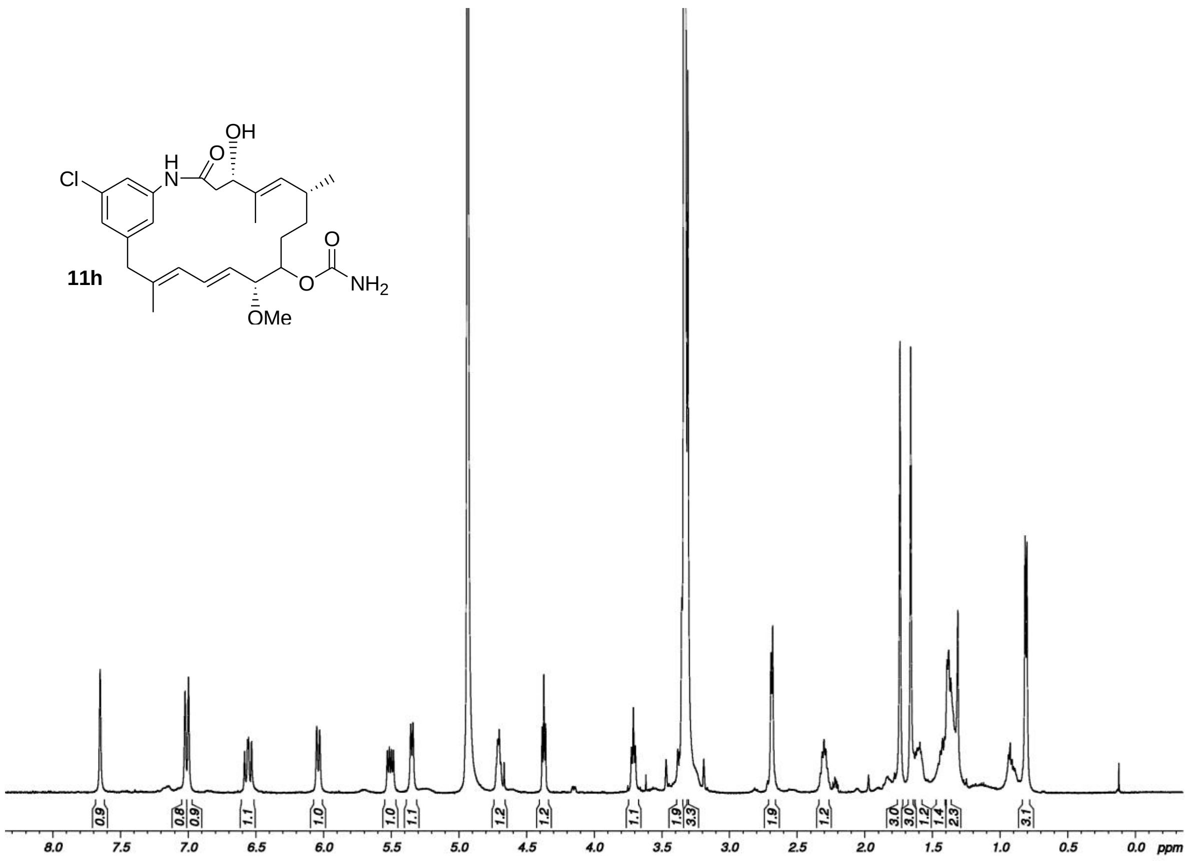
OH
H
N
O
Cl
11h
O
O
NH2
OMe
0.9
0.8
0.9
1.1
1.0
1.0
1.1
1.2
1.2
1.1
1.9
3.3
1.9
1.2
3.0
3.0
1.2
1.4
2.3
3.1
8.0 7.5 7.0 6.5 6.0 5.5 5.0 4.5 4.0 3.5 3.0 2.5 2.0 1.5 1.0 0.5 0.0 ppm

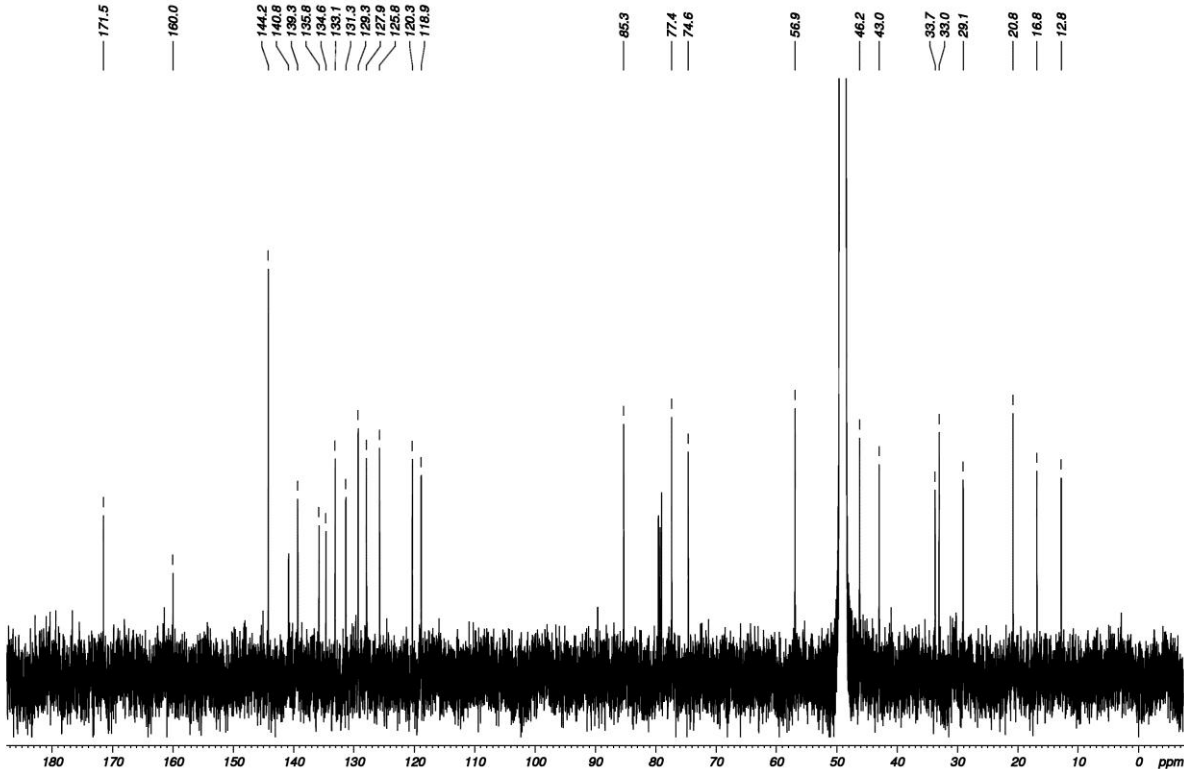
171.5
160.0
144.2
140.8
139.3
135.8
134.6
133.1
131.3
129.3
127.9
125.8
120.3
118.9
85.3
77.4
74.6
56.9
46.2
43.0
33.7
33.0
29.1
20.8
16.8
12.8
180 170 160 150 140 130 120 110 100 90 80 70 60 50 40 30 20 10 0 ppm

5. References

S1 Gottlieb, H. E.; Kotlyar, V.; Nudelman, A. *J. Org. Chem.* **1997**, *62*, 7512–7515. doi: 10.1021/jo971176v

S2 Leonard, J.; Lygo, B.; Procter, G. *Praxis in der organischen Chemie*; VCH, Weinheim, 1996.

S3 Knobloch, T.; Harmrolfs, K.; Taft, F.; Thomaszewski, B.; Sasse, F.; Kirschning, A. *ChemBioChem* **2011,** *12,* 540–547. doi:10.1002/cbic.201000608

S4 Eichner, S.; Knobloch, T.; Floss, H. G.; Fohrer, J.; Harmrolfs, K.; Hermane, J.; Schulz, A.; Sasse, F.; Spiteller, P.; Taft, F.; Kirschning, A. *Angew. Chem.* **2012,** *124,* 776–781, 10.1002/ange.201106249; Angew. Chem. Int. Ed. 2012, 51, 752–757. doi:10.1002/anie.201106249

S5 Moss, S. J.; Bai, L.; Toelzer, S.; Caroll, B. J.; Mahmud, T.; Yu, T.-W.; Floss, H. G. *J. Am. Chem. Soc.* **2002,** *124,* 6544–6545. doi:10.1021/ja020214b
